# A plant viral effector subverts FER‐RALF1 module‐mediated intracellular immunity

**DOI:** 10.1111/pbi.70099

**Published:** 2025-04-20

**Authors:** Penghuan Rui, Zhaoxing Jia, Xinxin Fang, Tianqi Yu, Wenqi Mao, Jiajia Lin, Hongying Zheng, Yuwen Lu, Feng Yu, Jianping Chen, Fei Yan, Guanwei Wu

**Affiliations:** ^1^ College of Life Sciences Fujian Agriculture and Forestry University Fuzhou China; ^2^ State Key Laboratory for Quality and Safety of Agro‐products, Key Laboratory of Biotechnology in Plant Protection of MARA, Key Laboratory of Green Plant Protection of Zhejiang Province, Institute of Plant Virology Ningbo University Ningbo China; ^3^ State Key Laboratory of Chemo/Biosensing and Chemometrics, College of Biology, Hunan Key Laboratory of Plant Functional Genomics and Developmental Regulation Hunan University Changsha China

**Keywords:** plant virus, turnip mosaic virus, *Nicotiana benthamiana*, FERONIA, rapid alkalinization factor, intracellular immunity

## Abstract

The receptor‐like kinase FERONIA (FER) is a prominent member of the *Catharanthus roseus* RLK1 (CrRLK1L) family, functioning as a modulator of immune receptor kinase complex formation in response to rapid alkalinization factors (RALFs). Typically, FER recognizes mature extracellular RALFs to combat bacterial and fungal infections. However, any role of the FER‐RALF signalling cascade in plant viral infections remains unexplored. Here, we used turnip mosaic virus (TuMV), an important member of the genus *Potyvirus*, and the host *Nicotiana benthamiana* as a model system to explore the role of the FER‐RALF cascade in plant–virus interactions. RALF1 from *N. benthamiana* (NbRALF1) positively regulated host resistance to inhibit TuMV infection. Co‐expression studies showed that this process does not involve the conserved RRXL and YISY motifs typically associated with RALF function. Instead, NbRALF1 induced cell death and significantly inhibited TuMV infection in a manner that depends on the entire RALF1 sequence and also NbFER. These results suggest a novel mechanism where NbRALF1 may inhibit viral infection through intracellular interactions with NbFER, differing from the previously reported extracellular FER‐RALF interactions that induce resistance to fungi and bacteria. Furthermore, we discovered that TuMV 6K2 interacts with NbRALF1 and promotes its degradation through the 26S proteasome pathway, thereby counteracting the host resistance induced by the NbFER‐NbRALF1 cascade. Our findings imply the existence of an uncharacterized intracellular immunity signalling pathway mediated by the NbFER‐NbRALF1 cascade and reveal a mechanism by which plant viruses counteract RALF1‐FER module‐mediated immunity.

## Introduction

Cell‐to‐cell communication is essential for plants, as they are sessile organisms that must adapt to a constantly changing environment. Plant peptide hormones play a pivotal role in this process, with the secreted, small (approximately 5 kDa) rapid alkalinization factor (RALF) peptides being particularly significant (Murphy *et al*., 2012). RALFs belong to a family of cysteine‐rich plant peptide hormones and play important roles in many physiological and developmental processes, ranging from plant reproduction and development to the modulation of immune responses (Abarca *et al*., 2021; Cheung, 2024; Haruta *et al*., 2014; Stegmann *et al*., 2017).

RALFs were first discovered because they induce moderate alkalinization in *Nicotiana tabacum* cell cultures (Pearce *et al*., 2001). They are now known to be widespread among terrestrial plants, with the majority of functional studies focusing on the model plant Arabidopsis, as well as on crops like rice (*Oryza sativa*), wheat (*Triticum aestivum*), maize (*Zea mays*), soybean (*Glycine max*), and rape (*Brassica rapa*). In *Arabidopsis thaliana*, over 30 RALF peptides have been identified (Abarca *et al*., 2021). Despite low amino acid similarity, RALF peptides share diverse conserved motifs that are crucial for their functions. These motifs include: (i), the dibasic RR motif, which is important for the proteolytic cleavage of the peptide precursor (Matos *et al*., 2008; Srivastava *et al*., 2009); (ii) the N‐terminal YI/LSY motif of the mature peptide, which is important for receptor binding and alkalinization activity (Pearce *et al*., 2010; Xiao *et al*., 2019); (iii) the four conserved cysteine residues in the C‐terminal region of RALFs, which form disulphide bonds and are essential for the peptide's bioactivity (Pearce *et al*., 2001).

RALFs are typical exocytosis proteins that undergo maturation through a specific cleavage at the N‐terminus RRXL site by proteolytic enzymes within the Golgi apparatus (Matos *et al*., 2008; Srivastava *et al*., 2009). This process is crucial for the activation of RALFs. In Arabidopsis, Site‐1‐Protease (S1P) plays a key role in this maturation process (Srivastava *et al*., 2009; Stegmann *et al*., 2017). Once mature, RALFs are released into the extracellular apoplast, where they can interact with their receptors, such as FERONIA (FER), a well‐known receptor kinase belonging to the *Catharanthus roseus* RLK1 (CrRLK1) family (Stegmann *et al*., 2017; Xiao *et al*., 2019). FER has an extracellular ligand‐binding domain (ECD), followed by a single transmembrane helix and a cytoplasmic kinase domain (CD) that is relatively well‐conserved. The general model suggests that FER is capable of sensing and recognizing RALF via its ECD, which then triggers phosphorylation of the CD and subsequently recruits specific intracellular substrates to initiate downstream signalling cascades (Li *et al*., 2016; Stegmann *et al*., 2017).

The RALF‐FER module functions by various mechanisms, including calcium signalling, hormone interactions, and production of reactive oxygen species (ROS) (Cheung, 2024; Guo *et al*., 2018; Kwon *et al*., 2024), playing a significant role in plant–microbe interactions. For example, the RALF23‐FER module in Arabidopsis suppresses the assembly of the bacterial molecular pattern flagellin‐induced immune complex EFR‐FLS2‐BAK1, thereby inhibiting the transmission of downstream immune signals like the ROS burst and mitogen‐activated protein kinase (MAPK) cascade, and enhancing the sensitivity of Arabidopsis to *Pseudomonas syringae* pv. tomato DC3000 (pst DC3000) (Stegmann *et al*., 2017). AtRALF23 can also suppress AtFER‐mediated MYC2 phosphorylation, thereby stabilizing MYC2 and enhancing JA signalling to negatively regulate plant immunity to pst DC3000 infection (Guo *et al*., 2018). More recently, a pioneering work revealed that upon detection of high bacterial colonization, mature RALF23 accumulates and activates the Zinc metalloproteinase At2‐MMP to trigger FER cytoplasmic domain cleavage, resulting in FERN. This cleaved fragment accumulates in the nucleus and enhances immunity‐related gene expression in a restricted region of the roots to protect against microbial invasion (Chen *et al*., 2024). Similarly, AtRALF22 plays a direct and positive role in immune responses, eliciting a variety of typical immune responses in a FER‐dependent manner to enhance Arabidopsis resistance against *Sclerotinia sclerotiorum* (He *et al*., 2023). The role of RALF1‐FER complexes in plant–microbe infection has rarely been investigated. RALF1 can act with FER to phosphorylate glycine‐rich RNA binding protein 7 (GRP7) to elevate GRP7 nuclear accumulation and trigger a rapid and massive RNA alternative splicing (AS) in *A*. *thaliana* (Wang *et al*., 2020). AS has been reported to play important roles in plant viral, bacterial, and fungal infections (Betz *et al*., 2024; Yang *et al*., 2014). Moreover, AtRALF1 promotes AtFER‐mediated phosphorylation of eIF4E1, a eukaryotic translation initiation factor that plays a crucial role in the control of mRNA translation rates, thereby increasing mRNA affinity and protein synthesis (Zhu *et al*., 2020). It is noteworthy that eIF4E1 can protect the translational machinery during turnip mosaic virus (TuMV) infection and restrict virus accumulation in Arabidopsis (Zafirov *et al*., 2023). These findings suggest that the FER‐RALF module plays complex roles in regulating plant immunity and microbial infections.

Moreover, some plant microbes secrete RALF‐like peptides that bind to the host's FER, facilitating their infection. Fungal pathogens, such as *Fusarium oxysporum* and the root‐knot nematode *Meloidogyne incognita*, secrete peptides that mimic plant RALFs and suppress host immunity (Masachis *et al*., 2016; Thynne *et al*., 2017; Zhang *et al*., 2020). In addition, the *F*. *graminearum* transcription factor FgPacC activates the expression of FgRALF, which binds to the wheat FER, suppressing plant immunity and promoting fungal infection (Wang *et al*., 2024a).

While the role of the RALF‐FER module in plant virus infection remains largely unexplored, the function of small secreted peptides in plant defence responses to viruses is increasingly recognized. For example, the potato (*Solanum tuberosum*) *PIP1* gene, predicted to encode a member of the pathogen‐associated molecular pattern (PAMP)‐induced peptide (PIP) family, triggers plant defence responses and confers resistance to potato virus Y infection (Combest *et al*., 2021). Additionally, the virus‐induced small peptide VISP1 acts as a selective autophagy receptor, targeting components of both antiviral RNA silencing and viral suppressors of RNA silencing (VSRs), thereby enhancing antiviral immunity (Tong *et al*., 2021, 2023). The plant small peptide CLAVATA3/ESR‐RELATED 7 (CLE7) can activate broad‐spectrum disease resistance to multiple RNA viral infections in *Nicotiana benthamiana* (Liu *et al*., 2024). To survive the ongoing “arms race” with their host plants, plant viruses often hijack host machinery to counteract defence mechanisms, and the detailed mechanisms behind these interactions require further investigation.

The genus *Potyvirus*, belonging to the family *Potyviridae*, is the largest group of known plant‐infecting RNA viruses, including many agriculturally important viruses such as turnip mosaic virus (TuMV; *Potyvirus rapae*) (Wu *et al*., 2024a; Yang *et al*., 2021). TuMV infects a wide variety of both cultivated crops and wild plants, including at least 318 species across 156 genera and 43 plant families (Mäkinen *et al*., 2023). The genome of TuMV has approximately 10,000 nucleotides, encoding 11 functional proteins. Among these, the second 6‐kDa protein, 6K2, is an integral membrane protein that plays crucial roles in remodelling the endoplasmic reticulum for the formation of viral replication vesicles (Wei *et al*., 2010; Wu *et al*., 2024b). Additionally, 6K2 is reported to enter the apoplast and facilitate viral long‐distance movement (Movahed *et al*., 2019). Notably, 6K2 is the only TuMV protein that triggers the unfolded protein response, thereby facilitating TuMV infection (Zhang *et al*., 2015).

*N*. *benthamiana* is a favoured experimental and model host because it is susceptible to a broad spectrum of plant viruses (Goodin *et al*., 2008). Here, we explored the role of the RALF‐FER module in plant virus infection and used TuMV‐*N. benthamiana* as a model system. We chose to study two *N. benthamiana* RALFs (NbRALF1 and NbRALF23), homologues of two well‐studied Arabidopsis RALFs. We provide evidence that NbRALF1, but not NbRALF23, participates in TuMV infection. NbRALF1 co‐operates with NbFER to trigger robust host immunity responses and combat TuMV infection, and this process is dependent on intracellular processes. We further discovered that TuMV protein 6K2 directly targets NbRALF1 and degrades it through the 26S proteasome pathway to counteract the NbRALF1‐NbFER module‐mediated host defence. These findings expand our understanding of the RALF‐FER signalling cascade from solanaceous plants in modulating plant immunity, and highlight the ongoing arms race between viruses and plant peptide hormones.

## Results

### NbRALF1 negatively regulates TuMV infection

We first investigated the role of NbRALF1 and NbRALF23 in TuMV infection by transiently co‐expressing each of them with a recombinant TuMV infectious clone, TuMV‐GFP, in *N. benthamiana* leaves. A 600‐bp fragment of the *GUS* (β‐glucuronidase) gene, designated as GUS^600^, was used as a control. As shown in Figure S1, treatment with NbRALF1‐myc attenuated the GFP fluorescence intensity resulting from TuMV‐GFP infection compared to the control (Figure S1a). Further analysis using Western blotting and RT‐qPCR revealed that both the viral coat protein and RNA accumulation were reduced when NbRALF1 was overexpressed (Figure S1b, c). The full‐length NbRALF1‐myc and its mature form of the predicted protein size were also detected (Figure S1b). In contrast, NbRALF23 had no effect on TuMV infection (Figure S1d–f). Thus, we focused on NbRALF1 for subsequent studies.

Upon TuMV infection, *NbRALF1* expression was continuously induced from 3 to 7 days post rub‐inoculation compared to the mock control (Figure S2). We then employed an intron‐spliced hairpin RNA‐mediated RNA interference approach to downregulate *NbRALF1* expression in the host plants. Artificial RNA silencing constructs containing either a partial *GUS* hairpin RNA (hpGUS) or a *NbRALF1* hairpin RNA (hpNbRALF1) were expressed in different regions of the same *N. benthamiana* leaves along with TuMV‐GFP. The regions treated with hpNbRALF1 exhibited substantial GFP fluorescence compared to those treated with hpGUS at 3 dpai. Western blot and RT‐qPCR analysis confirmed these observations (Figure S3a–c). RT‐qPCR data indicated that *NbRALF1* expression in the regions treated with hpNbRALF1 was reduced to approximately 40% of that in the control hpGUS (Figure S3c). Moreover, systematic silencing of *NbRALF1* using tobacco rattle virus showed developmental defects (Figure S4), and there was increased TuMV accumulation in *NbRALF1*‐silenced plants compared to the control (Figure S5a–c).

**Figure 1:**
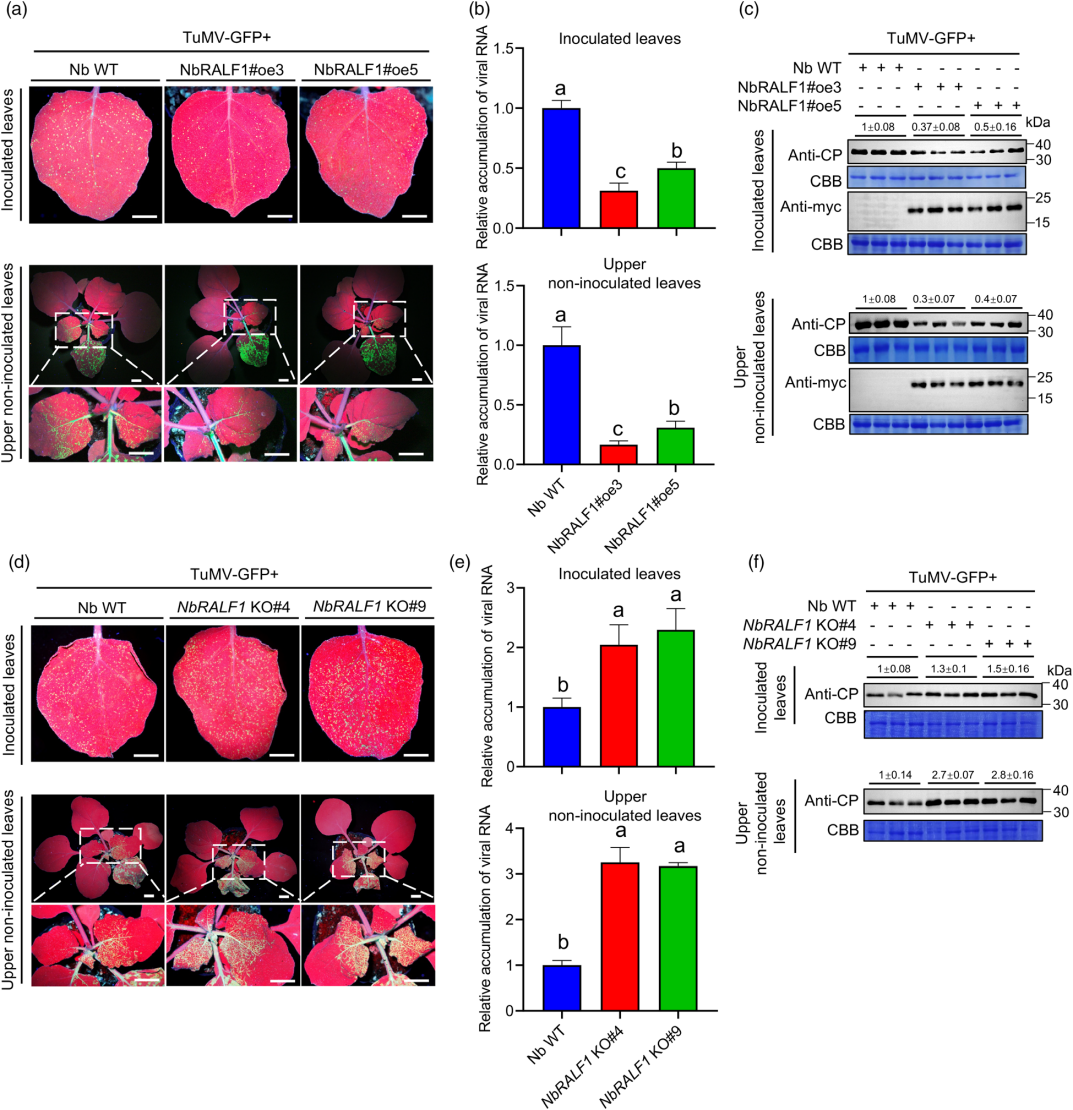
NbRALF1 negatively regulates TuMV infection. (a) GFP fluorescence of TuMV‐GFP infection under UV light, (b) RT‐qPCR analysis of TuMV RNA levels, and (c) Western blot analysis of TuMV CP and RALF1‐myc accumulation levels, in the local inoculated leaves (upper panels) and upper non‐inoculated leaves (lower panels) of wild‐type (WT) and RALF1oe *N. benthamiana* plants at 3‐ and 6‐days post‐inoculation (dpi). (d) GFP fluorescence of TuMV‐GFP infection under UV light, (e) RT‐qPCR analysis of TuMV RNA levels and (f) Western blot analysis of TuMV CP accumulation levels, in the local inoculated leaves (upper panels) and upper non‐inoculated leaves (lower panels) of WT and *RALF1*‐knockout (KO) *N. benthamiana* plants at 3‐and 6‐dpi. In panels (a) and (d), scale bar: 1 cm. For data in panels (c) and (f), relative TuMV CP band intensities were quantified by ImageJ software. Coomassie Brilliant Blue (CBB) R‐250‐stained RuBisco large subunit served as a loading control. For data in panels (b) and (e), error bars indicate mean ± SD (*n* = 5 independent plants). Statistical analysis was performed using One‐way ANOVA with Tukey's test; letters a‐c represent statistically different groups (*P* < 0.05).

To further confirm the role of NbRALF1 in TuMV infection, we generated transgenic lines overexpressing a NbRALF1‐myc fusion construct under the control of the cauliflower mosaic virus (CaMV) 35S promoter. Two independent T2 lines of NbRALF1 (NbRALF1oe#3 and NbRALF1oe#5) with relatively higher levels of the fusion proteins and transcripts were selected for further analysis (Figure S6a–c). These two NbRALF1oe lines were smaller than wild type (WT) plants (Figure S6a). After rub‐inoculation with TuMV, Western blot and RT‐qPCR results revealed that TuMV infection was consistently suppressed in both inoculated and upper non‐infected NbRALF1oe leaves compared to WT plants (Figure 1a–c). Two *NbRALF1*‐knockout (KO) *N. benthamiana* lines (NbRALF1 KO#4 and NbRALF1 KO#9) were also generated using CRISPR‐Cas9‐based technology. DNA sequencing results showed that each line had one inserted nucleotide at the cleavage site (Figure S7a). *NbRALF1* KO plants displayed a relatively slower growth phenotype than WT plants (Figure S7b). Consistent with previous observations, the TuMV infection assay on these plants showed increased GFP fluorescence intensity and viral accumulation levels compared to WT plants (Figure 1d–f). Moreover, infection assays with pepper mild mottle virus (PMMoV; *Tobamovirus capsici*, family *Vigaviridae*) and potato virus X (PVX; *Potexvirus ecspotati*, family *Alphaflexiviridae*) on these plants yielded comparable results to those obtained with TuMV (Figures S8 and S9). Together, these results indicate that NbRALF1 negatively regulates multiple virus infections. To be convenient, we chose TuMV as a model for further studies.

### NbRALF1 activates expression of a variety of immune response genes

**Figure 2:**
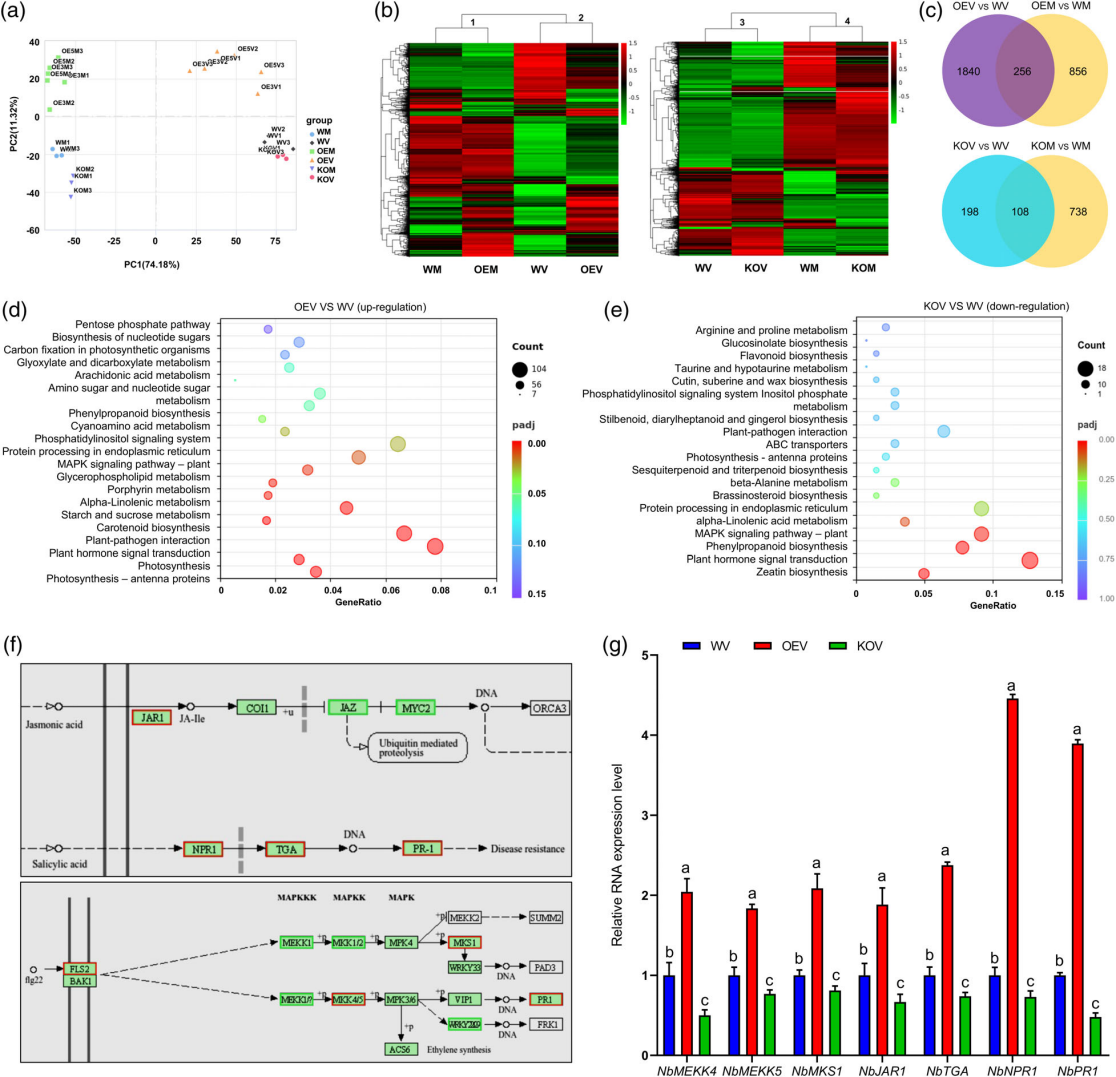
NbRALF1 activates the expression of a variety of immune response genes. (a) Principal component analysis (PCA) with k‐means clustering of the RNA‐seq results identified four clusters. The first capital letter(s) of the label stand for the two NbRALF1oe lines (OE3 and OE5) (OE), *NbRALF1* KO line (KO), or the WT *N. benthamiana* (W). The last one stands for the Mock treatment (M) or the TuMV treatment (V). The number on the label indicates the biological replicates. (b) A heatmap of differentially expressed genes (DEGs) between different samples. The heat map includes all genes that were differentially expressed in NbRALF1oe lines, *NbRALF1* KO line, and WT *N. benthamiana* seedlings under conditions of TuMV infection or mock treatment (|log2(fold change, FC)| > 1, adjusted *P*‐value (Padj) < 0.05). The columns are hierarchically clustered into three groups by the ward. The colour key represents Z‐Score transformation of the fold changes of all DEGs in the pairwise comparison group. Red indicates an increase in expression, green indicates a decrease in expression; colour intensity indicates the magnitude of the effect. (c) Venn diagram showing DEGs regulated by TuMV infection or Mock treatment in NbRALF1oe lines, *NbRALF1* KO line, or WT *N. benthamiana*. (d) KEGG pathway enrichment of the up‐regulated DEGs of NbRALF1oe plants in response to TuMV infection. (e) KEGG pathway enrichment of the down‐regulated DEGs of *NbRALF1* KO plants in response to TuMV infection. (f) Key DEGs in TuMV‐infected NbRALF1oe plants and *NbRALF1* KO plants compared to WT *N. benthamiana* enriched in pathways related to plant hormone signal transduction, MAPK signalling pathway, and plant‐pathogen interaction pathways following KEGG enrichment analysis. The DEGs are marked with red boxes. (g) RT‐qPCR analysis of the key DEGs expression levels. Error bars indicate mean ± SD (*n* = 5 independent plants). Statistical analysis was performed using one‐way ANOVA with Tukey's test; letters a‐c represent statistically different groups (*P* < 0.05).

To elucidate the mechanism by which NbRALF1 confers resistance to TuMV infection, we performed transcriptome profiling on two NbRALF1oe lines, the *NbRALF1* KO line and WT *N. benthamiana* seedlings under conditions of TuMV infection or mock treatment. The RNA‐seq data from all samples were categorized into four distinct clusters through principal component analysis (PCA) coupled with k‐means clustering. The first principal component (PC1) accounted for 74.18% of the variance, predominantly correlating with TuMV treatment. PC2 captured 11.32% of the variance and was largely associated with NbRALF1 overexpression or knockout (Figure 2a). We subsequently employed the clustering algorithm to discern the relationship between different genotypes and treatments by examining the expression of selected differential expressed genes (DEGs). As shown in Figure 2b, the samples segregated into four primary clusters based on the fold changes of selected DEGs, with cluster 1 and cluster 2 or cluster 3 and cluster 4 primarily distinguished by TuMV treatment, and inside each cluster primarily distinguished by *NbRALF1* overexpression or *NbRALF1* knock‐out. We then further analysed the RNA sequencing data to understand the transcriptional changes induced by *NbRALF1* overexpression and TuMV infection. A total of 1840 unique DEGs were identified in the OEV/WV comparison (TuMV‐treated NbRALF1oe *N. benthamiana* vs. TuMV‐treated WT *N. benthamiana*) relative to the OEM/WM comparison (mock‐treated NbRALF1oe *N. benthamiana* vs. mock‐treated WT *N. benthamiana*). Additionally, 198 unique DEGs were identified in the KOV/WV comparison (TuMV‐treated *NbRALF1* KO *N. benthamiana* vs. TuMV‐treated WT *N. benthamiana*) relative to the KOM/WM comparison (Figure 2c). KEGG pathway analysis of these unique DEGs revealed that the majority of up‐regulated DEGs in the OEV/WV comparison were implicated in plant hormone signal transduction, plant–pathogen interaction, protein processing in endoplasmic reticulum, and mitogen‐activated protein kinase (MAPK) signalling pathway (Figure 2d). Similarly, the majority of downregulated DEGs in the KOV/WV comparison were also implicated in these pathways (Figure 2e). We further examined the expression of DEGs in the terms ‘Plant hormone signal transduction’, ‘Plant‐pathogen interaction’, and ‘MAPK signalling pathway’. The JA biosynthesis gene *JAR1*, SA metabolism genes *NPR1*, *TGA*, and *PR‐1*, and plant immunity‐related genes *FLS2*, *MKS1*, *MKK4/5* and *PR1* were significantly upregulated in the OEV group and significantly downregulated in the KOV group, compared with WV (Figure 2f). RT‐qPCR confirmed that after TuMV infection, the expression of these genes was upregulated in the NbRALF1oe *N. benthamiana* but downregulated in the *NbRALF1* KO *N. benthamiana* (Figure 2g). Collectively, these findings suggest that NbRALF1 activates a variety of host defence pathways, thereby enhancing plant resistance to TuMV.

### NbRALF1‐mediated resistance depends on the entire RALF sequence, rather than the mature RALF1 domain

**Figure 3:**
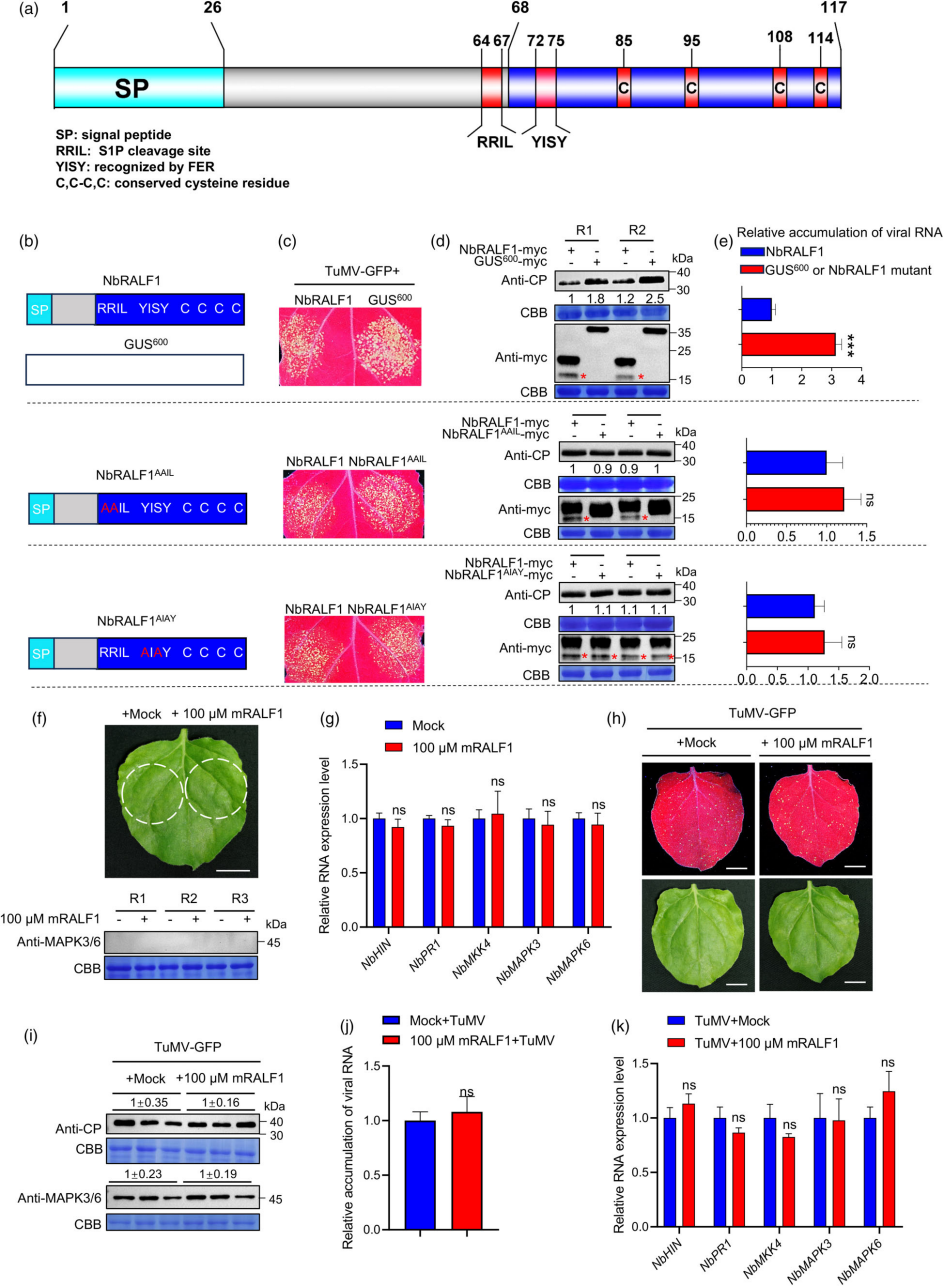
NbRALF1‐mediated host immunity activation depends on the entire RALF sequence, rather than the mature RALF1 domain. (a) Diagram showing the predicted conserved functional domains of NbRALF1. (b) Diagram showing the construction of NbRALF1 mutants. (c) GFP fluorescence of TuMV‐GFP infection in the inoculated leaves of *N. benthamiana* plants transiently co‐expressing TuMV‐GFP and NbRALF1 (left) or GUS^600^/NbRALF1^AAIL^/NbRALF1^AIAY^ (right) at 3 dpai under UV light. (d) Western blot analysis of protein accumulation levels from the samples in (c) at 3 dpai. R1 and R2 are two biological replicates. The mature form of NbRALF1 with expected band size is marked with red asterisks. (e) RT‐qPCR analysis of TuMV RNA levels from the samples in (c) at 3 dpai. (f) The effect of mRALF1 peptide spray application on MAPK3/6 activation. Samples were harvested for MAPK3/6 accumulation assay at 3 h post spray application. R1 to R3 are three biological replicates. (g) RT‐qPCR analysis of the immune‐related genes expression levels from the samples in (f). (h) GFP fluorescence of TuMV‐GFP infection in the mRALF1 peptide‐treated leaves of *N. benthamiana* at 3 dpi under UV light. Scale bar: 1 cm. (i) Western blot analysis of TuMV CP and MAPK3/6 accumulation levels from the samples in (h) at 3 dpi. (j) and (K) RT‐qPCR analysis of TuMV RNA and the immune‐related genes expression levels from the samples in (h) at 3 dpi. For data in panels (d), (f) and (h), relative TuMV CP and MAPK3/6 band intensities were quantified by ImageJ software. Coomassie Brilliant Blue (CBB) R‐250‐stained RuBisco large subunit served as a loading control. For data in panels (e), (g), (j) and (k), error bars indicate mean ± SD (*n* = 5 independent plants). Statistical analysis was performed using a two‐sided paired Student's *t*‐test (ns, non‐significant; ***, *P* < 0.001).

NbRALF1 is predicted to have a signal peptide sequence (1–26 aa) at its N‐terminus and a mature RALF domain (68–117 aa) at its C‐terminus, which includes four conserved cysteine residues (85, 95, 108, 114 aa). Additionally, NbRALF1 features the conserved RRIL (64–67 aa) and YISY (72–75 aa) motifs (Figure 3a). To determine the contribution of these motifs to RALF1‐mediated resistance against TuMV infection, we constructed a series of NbRALF1 mutants. These included: NbRALF1^1‐63^ and NbRALF1^68‐117^, which are truncated fragments expected to result from cleavage at the RRIL site; NbRALF1^AAIL^, where the S1P cleavage site RRIL was mutated to AAIL; NbRALF1^AIAY^, where the binding motif YISY in the mature RALF1, recognized by FER, was mutated to AIAY; and NbRALF1^C85A,C108A^, where the first and third cysteine residues were replaced with alanine (Figures 3b and S10a). Each mutant and the wild‐type RALF1 were co‐expressed with TuMV‐GFP in different patches of the same *N. benthamiana* leaf to assess any loss of resistance to TuMV infection. As shown in Figure S10b, both NbRALF1^1‐63^‐myc and NbRALF1^68‐117^‐myc displayed higher GFP fluorescence intensities compared to the normal NbRALF1‐myc. This suggests that NbRALF1‐mediated resistance does not rely on the mature RALF1 (NbRALF1^68‐117^‐myc) but rather on the entire RALF1. Moreover, the mutant NbRALF1^C85A,C108A^ showed similar phenotypes to these two mutants, indicating the essential role of cysteine residues in NbRALF1‐mediated resistance. Consistent with the above observations, the mutants that either abolished mature RALF1 production (NbRALF1^AAIL^) or disrupted FER recognition in the mature RALF1 (NbRALF1^AIAY^) exhibited similar fluorescent intensities to the wild‐type RALF1‐myc, suggesting that these functions may not be involved in NbRALF1‐mediated resistance (Figure 3b). The control GUS^600^‐myc consistently showed enhanced viral‐derived GFP fluorescence compared to NbRALF1‐myc. Western blotting and RT‐qPCR analysis confirmed these observations (Figure 3d,e; Figure S10c,d). Collectively, these results suggest that RALF1‐mediated resistance is dependent on the entire RALF1 sequence, particularly the conserved cysteine residues, rather than the previously reported mature RALF1 domain.

To further clarify the role of mature RALF1 in immunity activation and TuMV infection in *N. benthamiana*, we treated plant leaves with *in vitro* synthesized mature RALF1 peptide (mRALF1) via spray application. We monitored MAPK3/6 pathway activation, a key indicator of plant immunity, using a phosphorylation‐specific MAPK3/6 antibody. Western blot analysis revealed no detectable MAPK3/6 signal in either mRALF1‐treated or mock‐treated samples. Similarly, RT‐qPCR showed no significant difference in the expression of immune‐related genes between these treatments (Figure 3f,g). We then inoculated the treated leaves with TuMV‐GFP by rubbing the virus onto the leaf surface. As shown in Figure 3h–J, mRALF1 treatment did not affect TuMV infection levels compared to mock treatment. Notably, TuMV infection robustly activated the MAPK pathway in both treatment groups, producing comparable MAPK3/6 signals (Figure 3i). RT‐qPCR further confirmed that immunity‐related gene expression remained unchanged between mRALF1 and mock treatments (Figure 3k). Collectively, these results demonstrate that RALF1‐mediated resistance in *N. benthamiana* does not depend on the mature RALF1 domain.

### NbRALF1 relies on NbFER to trigger host immunity and suppress TuMV infection

**Figure 4:**
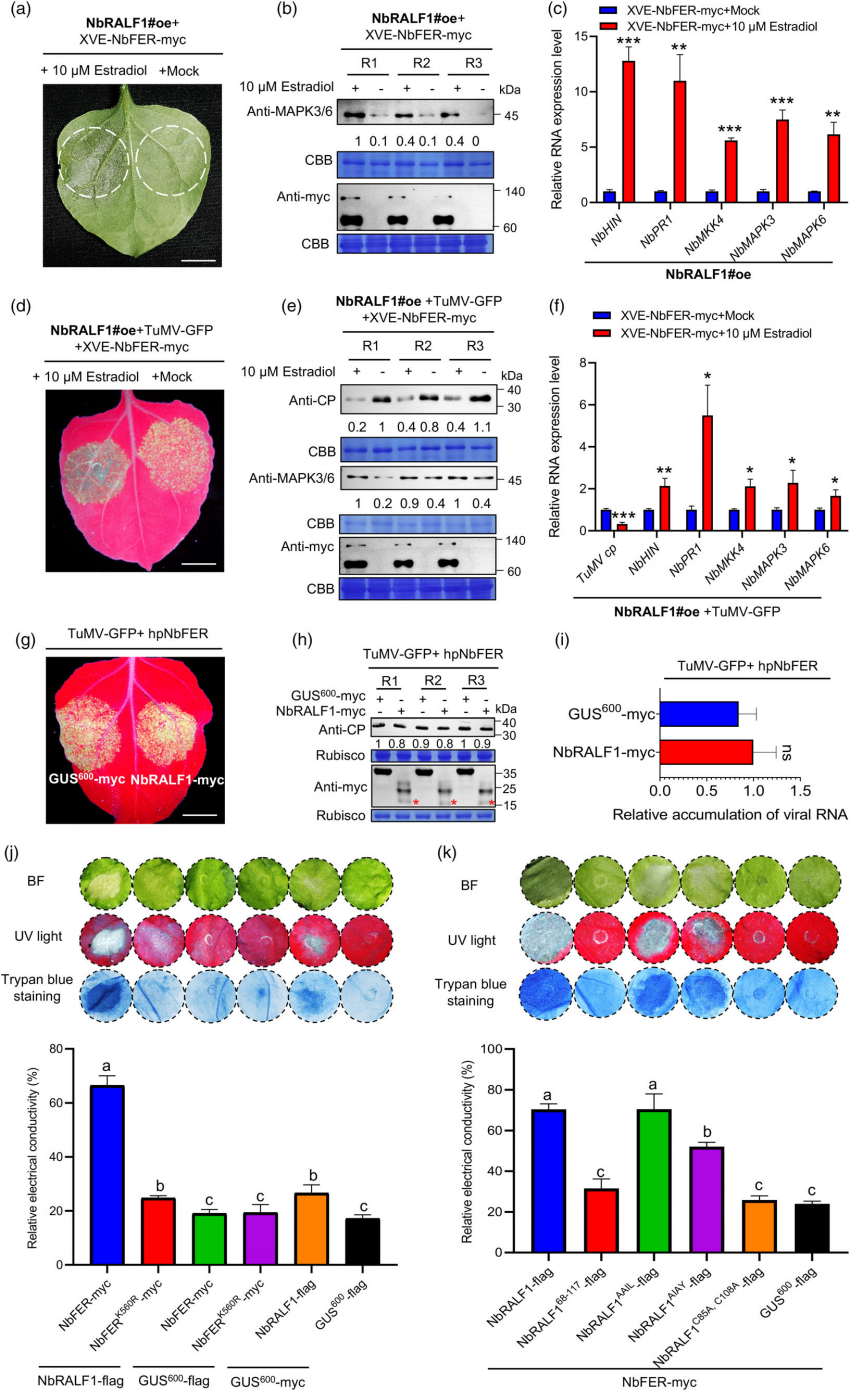
NbRALF1 relies on NbFER to trigger host immunity and suppress TuMV infection. (a) Inducible expression of XVE‐NbFER‐myc in NbRALF1oe plants. XVE‐NbFER‐myc was transiently expressed in NbRALF1oe *N. benthamiana* leaves. Infiltrated leaf areas were treated with 10 μM estradiol 24 h post‐agro‐infiltration for an additional 24 h. Scale bar: 1 cm. (b) Inducible expression of NbFER in NbRALF1oe plants enhanced MAPK3/6 expression. Western blot analysis determined MAPK3/6 and NbFER‐myc accumulation levels from the samples in (a). (c) RT‐qPCR analysis of the immune‐related genes expression levels from the samples in (a). (d) Inducible expression of NbFER in NbRALF1oe plants enhanced plant resistance to TuMV infection. GFP fluorescence of TuMV‐GFP infection in the inoculated leaves of NbRALF1oe *N. benthamiana* plants co‐expressing TuMV‐GFP and XVE‐NbFER‐myc at 3 dpai under UV light. Infiltrated leaf areas were treated with 10 μM estradiol 24 h post‐agro‐infiltration. Scale bar: 1 cm. (e) Western blot analysis of TuMV CP, MAPK3/6, and NbFER‐myc accumulation levels from the samples in (d) at 3 dpai. (f) RT‐qPCR analysis of TuMV RNA and the immune‐related genes expression levels from the samples in (d) at 3 dpi. (g) GFP fluorescence of TuMV‐GFP infection in the *NbFER*‐silenced leaves of *N. benthamiana* plants transiently co‐expressing TuMV‐GFP, hairpin RNA of NbFER (hpNbFER) along with GUS^600^‐myc (left) or NbRALF1‐myc (right) at 3 dpai under UV light. The constructs expressing hpNbFER were pre‐agroinfiltrated one day in advance. (h) Western blot analysis of protein accumulation levels from the samples in (g) at 3 dpai. The mature form of NbRALF1 with expected band size is marked with red asterisks. (i) RT‐qPCR analysis of TuMV RNA levels from the samples in (g) at 3 dpai. (j) and (k) Induction of cell death in *N. benthamiana* leaf tissues co‐expressing different constructs as indicated. Photographs were captured under bright light and UV light at 3 dpai. Cell death was observed by staining with trypan blue and quantified by measuring electrolyte leakage. For data in panels (b), (e), and (h), R1 to R3 are three biological replicates. Relative TuMV CP and MAPK3/6 band intensities were quantified by ImageJ software. Coomassie Brilliant Blue (CBB) R‐250‐stained RuBisco large subunit served as a loading control. For data in panels (c), (f), and (i), error bars indicate mean ± SD (*n* = 5 independent plants). Statistical analysis was performed using two‐sided paired Student's *t*‐test (ns, non‐significant; *, *P* < 0.05; **, *P* < 0.01; ***, *P* < 0.001). For data in panels (j) and (k), statistical analysis was performed using One‐way ANOVA with Tukey's test; letters a‐c represent statistically different groups (*P* < 0.05).

Next, we sought to investigate the involvement of NbFER in activating RALF1‐mediated immunity and resistance to TuMV infection. A co‐immunoprecipitation assay demonstrated that NbFER can indeed interact with NbRALF1 (Figure S11). Co‐expression of NbFER enhanced NbRALF1‐mediated resistance to TuMV infection (Figure S12a–c). This effect was found to be dependent on NbFER's kinase activity, as co‐expression of the kinase‐inactive mutant NbFER^K560R^ with NbRALF1 resulted in increased TuMV‐GFP infection (Figures S13 and S12d–f). In *N. benthamiana* protoplasts, co‐expression of NbFER and NbRALF1 significantly reduced viral RNA accumulation compared to NbRALF1 and GUS^600^ treatment (Figure S14a). Similarly, estradiol‐induced expression of NbFER (via XVE‐NbFER) in NbRALF1oe protoplasts suppressed viral RNA accumulation compared to mock‐treated samples (Figure S14b). Immunity‐related indicator assays showed that estradiol‐induced NbFER expression in NbRALF1oe plants increased MAPK3/6 accumulation and upregulated immune‐related genes (Figure 4a–c). Further TuMV infection assays revealed that estradiol‐induced NbFER expression reduced TuMV‐GFP infection and enhanced plant immunity in NbRALF1oe plants (Figure 4d–f). Notably, in *NbFER*‐silenced leaves, NbRALF1‐myc treatment lost its resistance to TuMV infection, showing similar viral accumulation to the GUS^600^‐myc treatment (Figure 4g–i). Collectively, these findings demonstrate that NbRALF1 requires the kinase activity of NbFER to confer resistance to TuMV infection.

While NbRALF1 expression can trigger a relatively weak cell death phenotype, co‐expression of NbFER with NbRALF1 induced much stronger cell death in *N. benthamiana* leaves, even in the absence of TuMV infection (Figure 4j). This was observable under both regular and UV light and was further confirmed through trypan blue staining and ion leakage assays (Figure 4j). Expression of NbFER with control did not result in this phenotype. Co‐expression of NbRALF1 with the kinase‐inactive mutant NbFER^K560R^ did not trigger cell death. Protein accumulation for each treatment was confirmed by Western blot analysis (Figure S15a). These results indicate that NbRALF1 co‐operates with NbFER and its kinase activity to trigger a robust host immunity response.

To further explore how the NbRALF1‐NbFER module activates host immunity, we investigated the effects of the NbRALF1 mutants as previously described. Wild‐type NbRALF1, each NbRALF1 mutant, and the GUS^600^ control were co‐expressed with NbFER in *N. benthamiana* leaves, and cell death phenotypes were assessed at 3 dpai. As shown in Figure 4k, when co‐expressed with NbFER, the mutant NbRALF1^AAIL^ showed a similar cell death phenotype to wild‐type NbRALF1, suggesting that this host immunity activation is not dependent on NbRALF1 maturation. Consistently, the mature NbRALF1^68‐117^ could not work together with NbFER to trigger cell death. Moreover, when the extracellular recognition motif YISY, recognized by NbFER, was mutated, the NbRALF1^AIAY^ treatment with NbFER still triggered cell death, although the phenotype was slightly milder compared to wild‐type RALF1, further supporting the idea that NbRALF1‐NbFER module‐mediated host immunity activation is largely independent of the extracellular reaction. Finally, the mutant NbRALF1^C85A,C108A^ could not work together with NbFER to trigger cell death. Protein accumulation for each treatment was confirmed by Western blot analysis (Figure S15b). When we individually expressed the wild‐type NbRALF1, each of its mutants, and the GUS^600^ control in leaves of *N. benthamiana*, none of these constructs induced cell death, with the exception of wild‐type NbRALF1, which resulted in a relatively weak cell death phenotype (Figure S16). Taken together, these results imply that while NbRALF1 relies on downstream NbFER to activate host immunity, the entire RALF1 and its conserved cysteine residues are crucial in this process.

### The intracellular RALF1‐FER cascade contributes to host immunity activation

**Figure 5:**
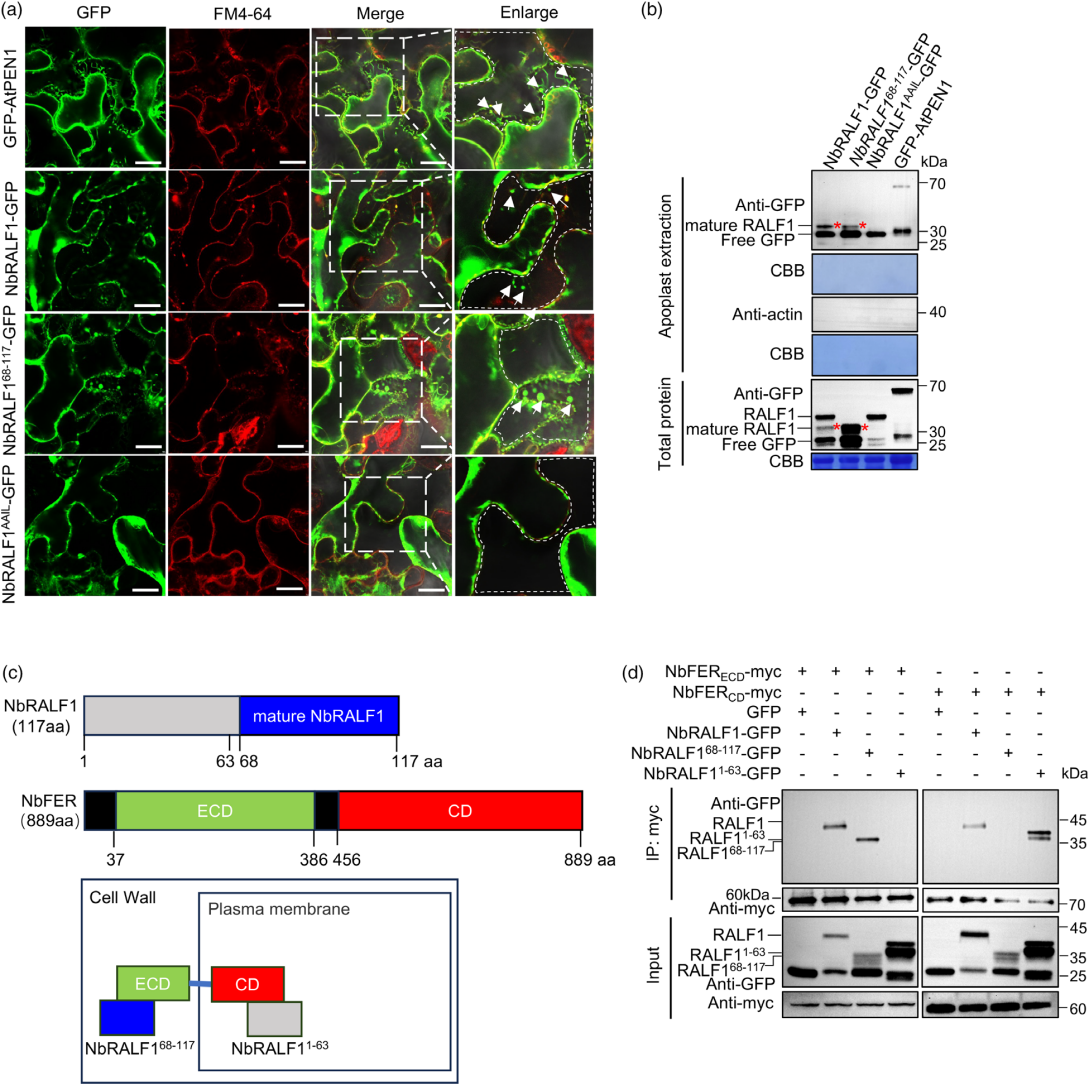
NbRALF1 can interact with NbFER intracellularly. (a) Plasmolysis assay for apoplast localization of NbRALF1 and its mutants NbRALF1^68‐117^ and NbRALF1^AAIL^. GFP‐AtPEN1, known to be able to localize to the apoplast, serves as a positive control. GFP‐AtPEN1, NbRALF1‐GFP, NbRALF1^68‐117^‐GFP, and NbRALF1^AAIL^‐GFP were transiently expressed in *N. benthamiana* leaves, which were then infiltrated with 10% NaCl at 2 dpai. Plasmolyzed cells in the leaf were stained with FM4‐64 and examined using confocal microscopy. Plasma membranes were stained with FM4‐64. Representative apoplast localization signals and regions are indicated with white arrows and dashed lines, respectively. Scale bar represents 20 μm. BF, bright field. (b) Western‐blot analysis with anti‐GFP antibodies of apoplastic fluid and total protein extractions from (a). The mature form of NbRALF1 with the expected band size is marked with red asterisks. (c) Schematic diagram of predicted NbFER interaction with NbRALF1. ECD, extracellular malectin‐like domain of NbFER. CD, intracellular kinase domain of NbFER. (d) Results of co‐IP assay showing that NbFER_ECD_ can form complexes with NbRALF1 and NbRALF1^68‐117^, while NbFER_CD_ can form complexes with NbRALF1 and NbRALF1^1‐63^, in *N*. *benthamiana* cells. Different cell lysates were immunoprecipitated with anti‐myc beads, separated by SDS‐PAGE, and immunoblotted with anti‐myc antibody or anti‐GFP antibody. Coomassie Brilliant Blue (CBB) R‐250‐stained RuBisco large subunit served as a loading control.

In Arabidopsis, mature RALF peptides bind to the ECD of FER, thereby activating FER kinase activity and subsequent downstream signalling cascades (Stegmann *et al*., 2017; Zhang *et al*., 2020). Given our observation that when expressed with NbFER, the mature NbRALF1^68‐117^ does not trigger cell death and that the NbRALF1^AAIL^ mutant, which expresses the entire RALF1 sequence but cannot be secreted to the apoplast (because the mutation prevents maturation) still elicits a cell death phenotype similar to that of wild‐type RALF1, we hypothesize that NbRALF1 may function intracellularly with NbFER to activate host immunity. To test this hypothesis, we first examined the subcellular localization of these mutants. The well‐characterized apoplast‐localizing protein AtPEN1 was used as a positive control (Movahed *et al*., 2019). Wild‐type NbRALF1, its mature form NbRALF1^68‐117^, and the NbRALF1^AAIL^ mutant were each fused with GFP at the C‐terminus and transiently expressed in *N. benthamiana* leaves. As shown in Figure 5a, the fluorescence of GFP‐AtPEN1 was observed between plasma membranes stained with FM4‐64 after plasmolysis, indicating apoplast localization. Both NbRALF1‐GFP and NbRALF1^68‐117^‐GFP were clearly visible in the apoplast, while the NbRALF1^AAIL^ mutant did not enter the apoplast, confirming its intracellular localization. We also extracted the apoplast components and validated these findings by immunoblotting analysis (Figure 5b). Meanwhile, we found that TuMV infection did not affect the localization of NbRALF1 and its mutants (Figure S17). Finally, BiFC and co‐immunoprecipitation (Co‐IP) assays provided additional evidence that the extracellular NbFER_ECD_ indeed binds to the mature NbRALF1 (residues 68–117), while the intracellular NbFER_CD_ was found to interact with the N‐terminal NbRALF1 (residues 1–63) (Figure S18; Figure 5c,d). Collectively, these findings provide further support for the notion that the capacity of NbRALF1 to activate host immunity may not require its secretion to the apoplast; instead, it could involve an intracellular interaction with NbFER in *N. benthamiana*.

### TuMV 6K2 and VPg interact with NbRALF1

**Figure 6:**
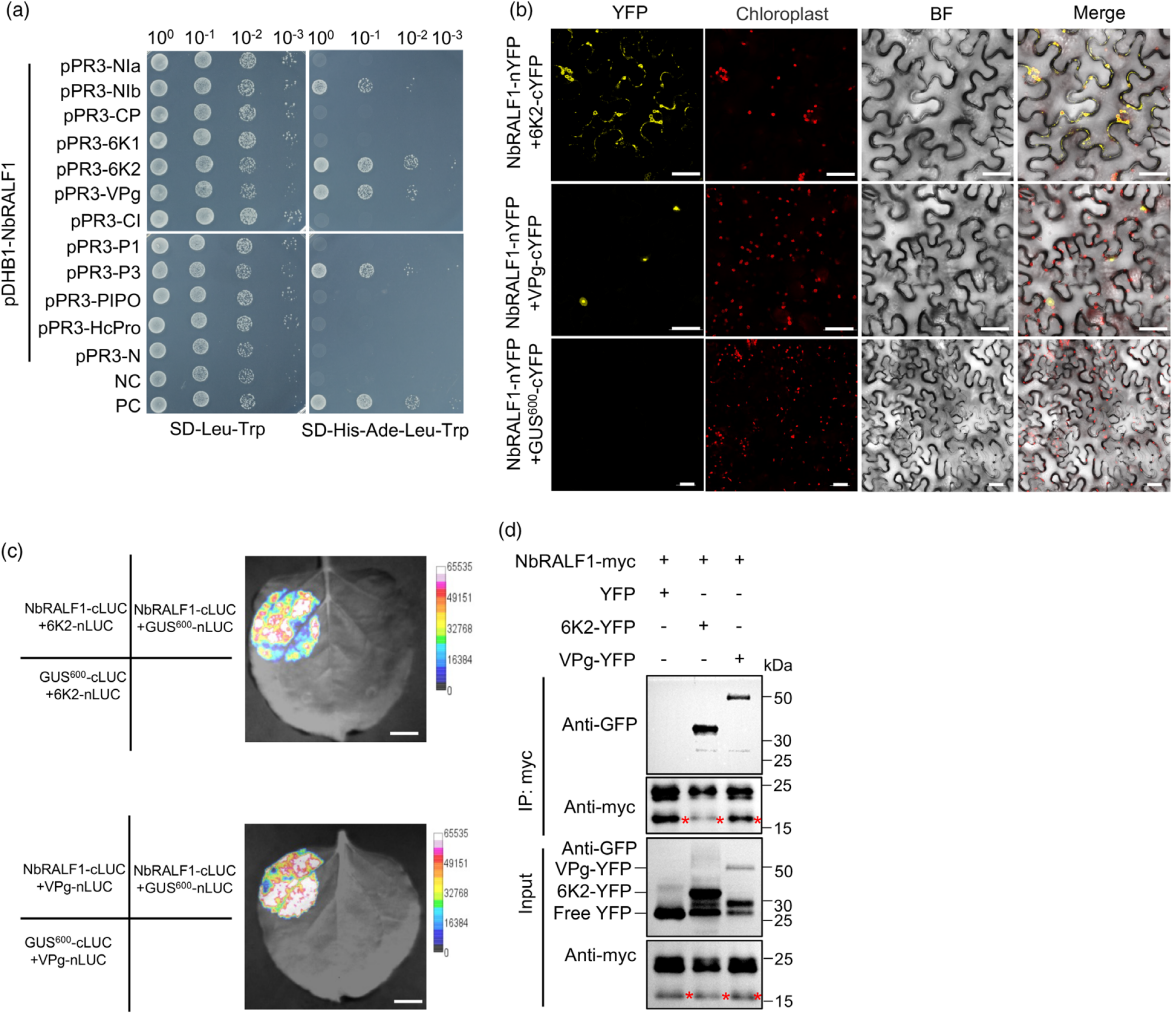
TuMV‐encoded 6K2 and VPg proteins interact with NbRALF1. (a) Protein–protein interaction assay between RALF1 and each of 11 TuMV proteins using a membrane yeast two‐hybrid (Y2H) method. NC, negative control. PC, positive control. (b) Images from a BiFC assay *in planta* to confirm the interactions detected by Y2H. The interactions between NbRALF1 and TuMV 6K2 or VPg were confirmed in *N*. *benthamiana* cells. Pictures were taken at 2 dpai. The YFP field and overlay of YFP with bright field (BF) and auto‐fluorescent chloroplasts are shown. Scale bar = 50 μm. (c) Split‐luciferase complementation imaging assay of the interaction between NbRALF1 and 6K2 or VPg. The pseudocolour bar indicates the range of luminescence intensity. (d) Results of co‐IP assay showing that NbRALF1 can form complexes with TuMV 6K2 and VPg in *N*. *benthamiana* cells. Different cell lysates were immunoprecipitated with anti‐myc beads, separated by SDS‐PAGE, and immunoblotted with anti‐myc antibody or anti‐GFP antibody. The mature form of NbRALF1 with expected band size is marked with red asterisks.

Given that TuMV can still establish successful infection in the presence of co‐expressed NbRALF1 and NbFER, without exhibiting an obvious cell death phenotype (Figure 4d), we hypothesized that TuMV may encode an effector that neutralizes the resistance conferred by the NbRALF1–NbFER module. As NbRALF1 functions upstream of NbFER, we have focused on possible protein–protein interactions between NbRALF1 and each of TuMV proteins. Yeast two‐hybrid (Y2H) analysis indicated potential interactions between NbRALF1 and several TuMV proteins, including VPg, P3, NIb, and 6K2 (Figure 6a). In subsequent BiFC assays, there were strong reconstituted YFP fluorescence signals indicating a positive interaction between NbRALF1 and the TuMV proteins 6K2 and VPg, but not the other viral proteins (Figure 6b; Figure S19). Negative controls exhibited no fluorescence signals. 6K2 interacted with NbRALF1 primarily in the cytoplasm and chloroplast, whereas the interaction between VPg and NbRALF1 appeared to be localized to the nucleus (Figure 6b). Luciferase complementation imaging (LCI) assays further validated the interactions between NbRALF1 and both 6K2 and VPg (Figure 6c). Finally, Co‐IP assays provided additional evidence that NbRALF1 can interact with both 6K2 and VPg (Figure 6d). Taken together, our findings conclude that TuMV 6K2 and VPg can directly interact with NbRALF1.

### TuMV 6K2 degrades NbRALF1 to counteract host immunity

**Figure 7:**
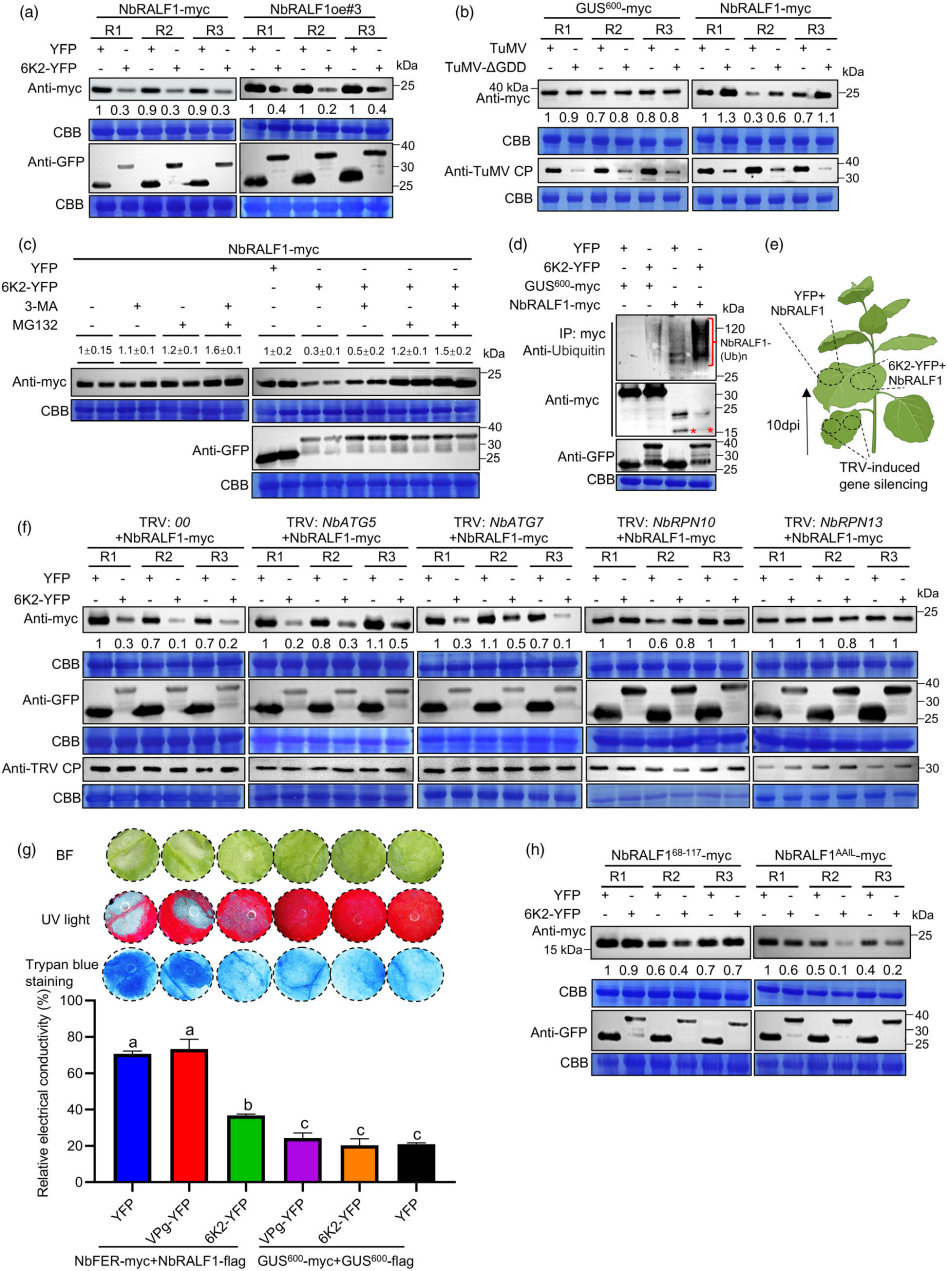
TuMV 6K2 degrades NbRALF1 intracellularly via the ubiquitination pathway and suppresses NbRALF1‐NbFER module‐triggered host cell death. (a) The effect of 6K2‐YFP overexpression on NbRALF1‐myc protein accumulation. YFP was used as a control. Left panels: NbRALF1‐myc was transiently co‐expressed with either YFP or 6K2‐YFP in two patches of the same wild‐type *N. benthamiana* leaf. Right panels: YFP and 6K2‐YFP were transiently expressed in two patches of the same *N. benthamiana* RALF1oe#3 leaf. Samples were harvested at 2 dpai for Western blotting assay with anti‐c‐myc and anti‐GFP monoclonal antibodies. (b) The accumulation of NbRALF1 in the context of TuMV infection. NbRALF1‐myc was transiently co‐expressed with TuMV or a replication‐deficit mutant TuMV‐ΔGDD in two patches of the same *N. benthamiana* leaf. GUS^600^‐myc served as a control. Samples were collected for Western blotting analysis at 3 dpai with anti‐c‐myc monoclonal antibody and anti‐TuMV CP polyclonal antibody. (c) The effect of autophagy inhibitor 3‐methyladenine (3‐MA) and 26S proteasome inhibitor carbobenzoxy‐L‐leucyl‐L‐leucyl‐L‐leucine (MG132) on NbRALF1 accumulation under treatment of 6K2‐YFP or YFP control. NbRALF1‐myc was transiently co‐expressed with 6K2‐YFP or YFP in *N. benthamiana* leaves and then the infiltrated leaf areas were treated with 100 μM 3‐MA, MG132, or both, at 40 h post‐agro‐infiltration for an additional 8 h. Total proteins from infiltrated regions were extracted and subjected to immunoblotting analysis using anti‐c‐myc and anti‐GFP monoclonal antibodies. (d) Western blot detection of endogenous ubiquitin with 6K2‐NbRALF1 complex in *N. benthamiana* leaves. 6K2‐YFP or YFP were transiently co‐expressed with NbRALF1‐myc or GUS^600^‐myc in *N. benthamiana* leaves. Different cell lysates were immunoprecipitated with anti‐myc beads, separated by SDS‐PAGE and immunoblotted with anti‐Ub and anti‐c‐myc monoclonal antibodies. Typical ubiquitin signals are shown. (e) Schematic representation of the experimental design for TRV‐induced gene silencing and transient expression treatment in upper silenced *N. benthamiana* leaves. (f) The effect of silencing of autophagy or ubiquitin pathway genes on 6K2‐mediated RALF1 degradation in *N. benthamiana*. (g) The effect of TuMV 6K2 or VPg expression on NbFER‐NbRALF1 modulate‐mediated cell death in *N. benthamiana*. Photographs were captured under bright light and UV light at 3 dpai. Cell death was observed by staining with trypan blue and quantified by measuring electrolyte leakage. Error bars indicate mean ± SD (*n* = 5 independent plants). Statistical analysis was performed using One‐way ANOVA with Tukey's test; letters a‐c represent statistically different groups (*P* < 0.05). (h) Effect of 6K2 treatment on the accumulation of mature NbRALF1^68‐117^ or NbRALF1^AAIL^. NbRALF1^68‐117^‐myc or NbRALF1^AAIL^‐myc was transiently co‐expressed with YFP and 6K2‐YFP, respectively, in two patches of the same *N. benthamiana* leaf. Samples were collected for Western blotting analysis at 2 dpai. For data in panels (a), (b), (c), (f) and (h), relative NbRALF1‐myc, GUS^600^‐myc, NbRALF1^68‐117^ and NbRALF1^AAIL^ band intensities were quantified by ImageJ software. Coomassie Brilliant Blue (CBB) R‐250‐stained RuBisco large subunit served as a loading control. R1 to R3 are three biological replicates.

In our Co‐IP assay, we observed a notable decrease in NbRALF1‐myc accumulation when it was co‐expressed with 6K2‐YFP, but not with VPg‐YFP or YFP control, in the input samples (Figure 6d). To further confirm this observation, we transiently co‐expressed NbRALF1 and YFP, 6K2‐YFP or VPg‐YFP in different patches of the same leaves of *N. benthamiana* plants. Western blotting results clearly showed that NbRALF1‐myc accumulation was reduced by 60%–80% under 6K2‐YFP treatment compared to the YFP control (Figure 7a). In contrast, VPg‐YFP treatment did not affect NbRALF1‐myc accumulation (Figure S20). We also transiently expressed YFP and 6K2‐YFP in different patches of the same leaves of NbRALF1oe#3 plants, and NbRALF1‐myc accumulation was consistently reduced under 6K2‐YFP treatment (Figure 7a). We further examined this phenomenon in the context of TuMV infection. A replication‐defective mutant TuMV‐ΔGDD served as a negative control. While GUS^600^‐myc accumulation remained unaffected under TuMV infection, NbRALF1‐myc levels decreased compared to those under TuMV‐ΔGDD treatment (Figure 7b). The minimal coat protein accumulation observed in TuMV‐ΔGDD‐treated samples was attributed to the activity of the 35S promoter (Figure 7b). To test the reverse relationship, we transiently co‐expressed 6K2‐YFP with NbRALF1‐myc or GUS^600^‐myc in different patches of the same *N. benthamiana* leaf. Western blot analysis revealed that 6K2‐YFP accumulation levels remained unchanged under NbRALF1‐myc treatment compared to the GUS^600^‐myc control treatment (Figure S20).

To explore the potential mechanism underlying 6K2‐mediated NbRALF1 degradation, we treated the samples with carbobenzoxy‐L‐leucyl‐L‐leucyl‐L‐leucinal (MG132), which inhibits the 26S proteasome, or 3‐methyladenine (3‐MA), which inhibits autophagy, or both. Our results indicated that MG132 or both, but not 3‐MA alone, could alleviate 6K2‐mediated NbRALF1 degradation, resulting in accumulation levels comparable to those observed in the YFP control treatment (Figure 7c). These findings suggest that NbRALF1 turnover is primarily dependent on the ubiquitin (Ub)‐proteasome pathway. To confirm whether NbRALF1 is targeted for degradation by the 26S proteasome pathway, we performed a co‐IP experiment to detect the presence of endogenous Ub in the NbRALF1‐6K2 protein complex. As shown in Figure 7d, a large amount of Ub was detected with a Ub‐specific antibody in samples co‐expressing NbRALF1‐myc and 6K2‐YFP. In contrast, little to no Ub was detected in the control treatments.

Finally, we used a TRV‐based virus‐induced gene silencing system to silence key autophagy (*NbATG5* or *NbATG7*) or 26S proteasome (*NbRPN10* or *NbRPN13*) pathway genes (Ji *et al*., 2021) (Figure 7e; Figure S22), and then investigated their effects on NbRALF1 accumulation. While 6K2‐YFP readily reduced NbRALF1‐myc accumulation compared to the YFP control under TRV:00 treatment, silencing of either *NbRPN10* or *NbRPN13* substantially alleviated 6K2‐mediated NbRALF1 degradation. Silencing of the two autophagy pathway genes did not prevent 6K2‐mediated NbRALF1 degradation (Figure 7f). Taken together, these results provide compelling evidence that 6K2 targets NbRALF1 for degradation via the 26S proteasome pathway.

Subsequently, we investigated the effect of 6K2 and VPg on the cell death induced by the RALF1‐FER module in host cells. As shown in Figure 7g, treatment with 6K2 significantly mitigated the cell death triggered by the co‐expression of NbFER and NbRALF1. In contrast, VPg had no observable effect. This phenomenon was evident under both regular and UV light and was further validated through trypan blue staining and ion leakage assays. Western blotting analysis confirmed protein accumulation for each treatment (Figure S23). Finally, as TuMV 6K2 can also target the apoplast (Movahed *et al*., 2019), we determined whether 6K2 targeted NbRALF1 for degradation within cells or in the extracellular space. To this end, the mutant NbRALF1^AAIL^ or the mature form of NbRALF1 (NbRALF1^68‐117^) was co‐expressed with 6K2‐YFP or YFP in *N. benthamiana* leaves. We had earlier demonstrated that NbRALF1^68‐117^‐GFP was clearly visible in the apoplast, while the NbRALF1^AAIL^ mutant did not enter the apoplast (Figure 5). Here, Western blotting results revealed that when co‐expressed with 6K2‐YFP, the levels of NbRALF1^AAIL^‐myc significantly decreased compared to the YFP control, while the mature NbRALF1^68‐117^‐myc levels remained unaffected (Figure 7h). Taken together, these findings suggest that TuMV 6K2 targets NbRALF1 for intracellular degradation, thereby counteracting the activation of host immunity mediated by the NbRALF1‐NbFER module.

## Discussion

The RALF family of peptides, initially identified by an ability to cause rapid alkalinization of tobacco (*N*. *tabacum*) extracellular media and to activate the MAPK pathway (Pearce *et al*., 2001), has since been implicated in plant immunity. However, our understanding of its roles and mechanisms in this context remains incomplete. While RALFs are a widespread superfamily in plants, experimental evidence for their involvement in plant immunity has been limited to a few members, such as RALF17, RALF23, and RALF33, which have been shown to play a role against hemibiotrophic pathogens (Merino *et al*., 2019; Stegmann *et al*., 2017). It has not been clear whether RALFs have a role in plant virus infection. In this study, we provide the first evidence that NbRALF1 can negatively regulate a viral infection (Figure 1), unveiling a new facet of RALF involvement in plant–virus interactions.

Most mature RALF peptides in plants share conserved domains, including the YISY motif at the N terminus and four cysteine residues near the C terminus (Campbell and Turner, 2017). In Arabidopsis, the YISY motif of mature RALF peptides is known to bind to the FER ECD, initiating the FER kinase pathway (Haruta *et al*., 2014; Stegmann *et al*., 2017; Zhang *et al*., 2020). Our results indicate that NbRALF1 can induce plant basal immunity and suppress TuMV infection. However, this ability is independent of the conserved RRIL and YISY motifs of NbRALF1 (Figure 3). Retaining only the mature peptide (68–117 aa) or signal peptide (1–63 aa) of NbRALF1, or mutating its cysteine conserved site, all resulted in a loss of ability to inhibit TuMV infection (Figure S10). Spray application of mRALF1 also showed no resistance to TuMV infection (Figure 3h–j). The ability of NbRALF1 to inhibit TuMV infection is dependent on NbFER and its phosphorylation function (Figure 4; Figure S12). Co‐infiltration of NbRALF1 and NbFER induced a strong cell death response which was not abolished by mutations in the RRIL or YISY motifs of NbRALF1 (Figure 4). This suggests that NbRALF1 may recognize the intracellular portion of NbFER to induce a stronger immune response and suppress viral infection, differing from the PTI induced by YISY‐FER binding reported in responses to fungi and bacteria. Moreover, in a very recent study, AtFER was found to undergo cleavage at its cytoplasmic domain following bacterial invasion, and the resulting cleavage fragment, FERN, accumulated in the nucleus and enhanced the immune response in roots, thereby defending against bacterial infections (Chen *et al*., 2024). Intriguingly, we also detected a smaller fragment of FER that corresponds in size to FERN upon the co‐expression of NbFER and TuMV‐GFP (Figure S12b,e). It remains to be determined whether this fragment is a specific response to viral infection and what role it plays in the immunity response.

Potential RALF peptides have also been identified in fungal and bacterial genomes (Masachis *et al*., 2016; Thynne *et al*., 2017). For example, *F*. *oxysporum* secretes RALF‐like peptides that mimic plant RALFs, activate FER kinase, and inhibit AHA activity, inducing apoplast alkalinization and activating the orthologous MAPK FMK1 kinase, which promotes virulence in fungi (Masachis *et al*., 2016). However, plant virus genomes, which are generally small and encode limited proteins, do not seem to encode RALF‐like sequences. Our comparison of RALFs and TuMV genome sequences found no RALF‐like sequences in TuMV. This raises the question of how TuMV resists NbFER‐NbRALF1 module‐induced resistance to establish a successful infection. Further studies showed that TuMV‐encoded 6K2 protein can interact with NbRALF1 (Figure 6). Interestingly, this interaction inhibits the accumulation of NbRALF1 at the protein level (Figure 7). Further analysis revealed that 6K2 can degrade NbRALF1 through the 26S proteasome pathway (Figure 7). This suggests that, unlike fungi and bacteria, which can secrete RALF‐like proteins, viruses use their own encoded proteins to hijack the host proteasome pathway to suppress NbRALF1‐NbFER module‐induced immunity, thereby facilitating their own infection.

Our study also raises several questions that warrant further investigation. Firstly, the mechanisms by which NbRALF1 recognizes viral infection and transmits immune signals are not yet clear. It is possible that NbRALF1 can recognize TuMV VPg to mediate this process (Figure 6), but this requires further study. Secondly, while the YISY motif of mature RALF1 is known to regulate the PTI response by binding to the FER extracellular domain (Haruta *et al*., 2014; Stegmann *et al*., 2017; Zhang *et al*., 2020), our study suggests that NbRALF1 may recognize the intracellular domain of FER to induce a stronger immune response. The detailed mechanism by which this recognition induces cell death remains unclear. Additionally, the critical downstream signalling components involved in the NbRALF1‐NbFER module‐mediated host immunity activation require further investigation. It has been reported that eIF4E1 is required to maintain global plant translation and to restrict TuMV accumulation during infection (Zafirov *et al*., 2023), and AtRALF1 can promote AtFER‐mediated eIF4E1 phosphorylation (Zhu *et al*., 2020), so it is reasonable to assume that eIF4E1 may be a key target in the NbRALF1‐NbFER‐mediated resistance against TuMV infection.

**Figure 8:**
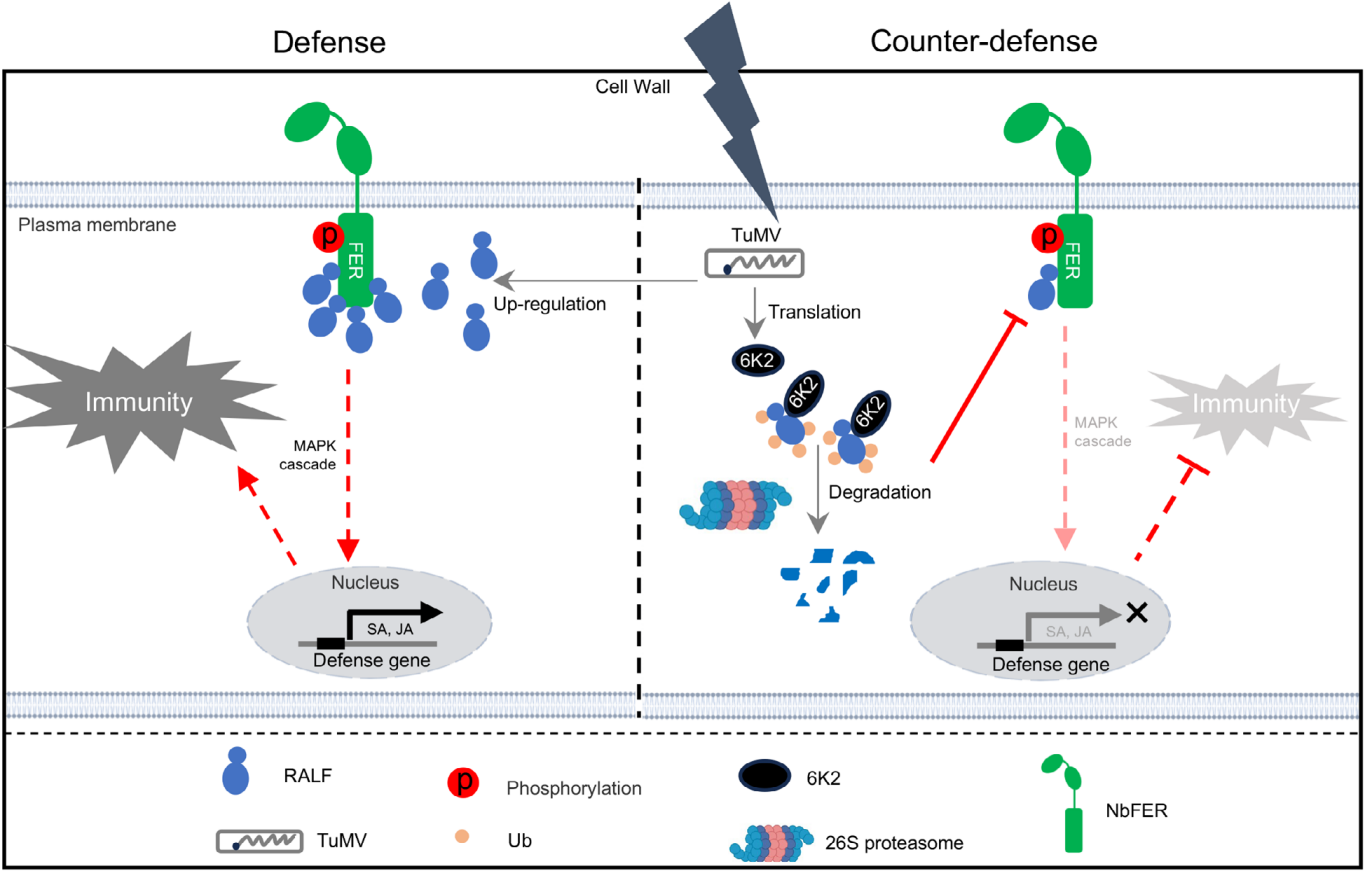
A proposed model: a plant viral effector suppresses NbFER‐NbRALF1‐mediated intracellular immunity in *N. benthamiana*. In the absence of TuMV infection, NbRALF1 likely recognizes the intracellular CD of FER to activate its kinase activity and subsequent initial downstream immune responses. Upon TuMV infection, *NbRALF1* is upregulated and co‐operates with NbFER to enhance the plant immunity response, probably by binding with the intracellular cytoplasmic domain of FER. To counteract this defence, TuMV‐encoded 6K2 targets NbRALF1 for degradation via the 26S proteasome pathway, thereby subverting the NbRALF1‐NbFER module‐mediated host immunity defence and finally establishing successful infection. The figure was partially created using BioRender.

In conclusion, we propose a model for the role of RALF1‐FER module in activating host immunity and the counteracting strategy of a plant virus (Figure 8): In the absence of TuMV infection, NbRALF1 likely recognizes the intracellular CD of FER to activate its kinase activity and subsequent downstream immune responses. Upon TuMV infection, *NbRALF1* is upregulated to enhance this immune response and defend against TuMV infection. To counteract this host defence response, TuMV‐encoded 6K2 protein directly interacts with NbRALF1 and promotes its degradation through the 26S proteasome pathway, thereby establishing a successful systemic infection.

## Materials and methods

### Plant materials and virus inoculation

Plants stably overexpressing the pCV‐NbRALF1‐myc construct or with a knock‐out of the *NbRALF1* gene were generated by leaf disk transformation using wild‐type *N. benthamiana* as the background line. Transformants were screened for hygromycin B resistance on MS medium. All *N. benthamiana* plants were grown in an insect‐free greenhouse at 24 ± 1 °C, with a light/dark cycle of 16 h/8 h and a relative humidity of 70 ± 5%, as described previously (Rui *et al*., 2022a).

### Plasmid construction

The coding sequences of genes of interest were amplified from *N*. *benthamiana* cDNA using PCR with PrimerSTAR Max DNA Polymerase (Takara, Japan), and then cloned into various expression vectors, employing either a ligation‐independent cloning method or Gateway cloning technology. Coding sequences of TuMV genes were amplified from the infectious clone TuMV::GFP (GenBank Accession number: NC_002509.2). Site‐directed mutagenesis of *NbRALF1* was achieved using overlap extension PCR with specific primer pairs listed in Table S1. The pEarleyGate104 plant binary expression vector was used to generate N‐terminal YFP‐tagged constructs (Earley *et al*., 2006). The pCV‐LIC‐Myc vector was used to construct C‐terminal myc‐tagged constructs. A β‐estradiol‐inducible Gateway‐compatible destination vector pMDC7 was used to express XVE‐NbFER‐myc fusion protein. The vectors pCV‐LIC‐nYFP and pCV‐LIC‐cYFP were used to produce constructs for bimolecular fluorescence complementation (BiFC) assays, while pCV‐LIC‐nLuc and pCV‐LIC‐cLuc were employed for the split‐luciferase assay (Fang *et al*., 2024). All plasmids were verified by DNA sequencing.

### Protein extraction and western blot analysis

Plant samples were harvested and processed by grinding them into a fine powder using liquid nitrogen. Protein lysis buffer, composed of 0.01 M Phosphate Buffered Saline (PBS) at pH 7.5, 2% sodium sulphite (Na_2_SO_3_), 0.05% NP‐40, and 0.1% β‐mercaptoethanol, along with SDS‐PAGE loading buffer, was added to the powdered samples and incubated in a metal bath at 85 °C for 15 min. The samples were then rapidly cooled on ice for 5 min before centrifugation at 12000 rpm for 5 min to separate the protein extracts. Chemiluminescent imaging was conducted using the Immobilon Western chemiluminescent horseradish peroxidase (HRP) substrate (Millipore) on an Amersham Imager 680 machine (GE), following the manufacturer's instructions. After the chemiluminescence detection on the PVDF membrane, Coomassie Brilliant Blue (CBB) staining was used to assess the loading consistency across different parallel gels.

### RNA isolation and quantitative reverse transcription PCR (RT‐qPCR)

EasyPure plant RNA kit (Transgen biotech, China) was used to extract total RNA from plant samples, following the manufacturer's instructions. TransScript All‐in‐One kit (Transgen biotech, China) was used to eliminate genomic DNA and synthesize the first‐strand cDNA from RNA extracts, which was used for subsequent qPCR. SYBR Green PCR master mix kit (Vazyme, China) was used to conduct qPCR tests on a LightCycler 480 II real‐time PCR system (Roche, Germany). The experimental data were analysed using the delta–delta C_T_ method to determine relative gene expression levels of the genes of interest. The gene *actin II* from *N. benthamiana* was selected as the internal reference control for normalization (Wu *et al*., 2022). Each independent experiment included biological triplicates, with a minimum of three technical replicates per sample. The specific primers used for qPCR in our study are detailed in Table S1.

### RNA‐seq treatment

In the *in vitro* RNA‐Seq experiment, three‐week‐old NbRALF1oe lines (OE3 and OE5), *NbRALF1* KO line, and WT *N. benthamiana* seedlings were inoculated with TuMV or 1× PBS (Mock). The upper non‐inoculated leaves of treated plant samples were collected 7 days after infiltration. Total RNA extraction, cDNA library construction, and sequencing were carried out by Novogene (Beijing) Co., Ltd. on a Novaseq‐PE150. High‐quality clean reads were obtained after filtration, and they were further aligned to the *N. benthamiana* reference genome using the genome Nb HZ version 1, available at https://lifenglab.hzau.edu.cn/Nicomics (Wang *et al*., 2024b). Bioinformatic analysis was performed using the Novomagic tools at https://magic‐plus.novogene.com. Experiments were carried out with three biological replicates for each treatment.

### Protoplast isolation and virus replication assay

Protoplast isolation was performed as previously described (Fang *et al*., 2024). Briefly, about 0.1 g of *N. benthamiana* leaf tissues (with the lower epidermis removed) was digested with 5 mL of enzyme mixture (containing 1.5% cellulase R‐10, 0.5% macerozyme R‐10, 5 mM 2‐morpholinoethanesulfonic acid (MES), 0.1% bovine serum albumin, 10 mM CaCl_2_, and 0.4 M mannitol, pH 5.8), and incubated at 25 °C for 1 h in the dark. After digestion, the protoplasts were collected from the interface of 0.4 M mannitol‐MES and 0.55 M sucrose solution by centrifugation. The purified protoplasts were then diluted appropriately, and their concentration was determined using a haemocytometer under a microscope. About 1 × 10^5^ protoplasts were transfected with indicated plasmids in 40% PEG 4000 in 0.8 M mannitol and 1 M CaCl_2_ at room temperature for 20 min. Transformed protoplasts were then washed and resuspended in W5 buffer (154 mM NaCl, 125 mM CaCl_2_, 5 mM KCl, and 2 mM MES, pH 5.7) and incubated for protein expression and virus replication.

### Trypan blue staining assay

Trypan blue staining was performed as previously described (Yuan *et al*., 2023). The trypan blue staining working solution consisted of 10 mL lactic acid, 10 mL sterile distilled water, 10 mL phenol, 10 mL glycerol, and 0.1 g trypan blue. Treated plant leaves were harvested with scissors at 3 dpai and then washed twice with sterile distilled water. The cleaned *N. benthamiana* leaves were then put in fresh trypan blue solution and boiled for 10 min, ensuring that the leaves were completely immersed in the liquid. After the working solution had cooled to room temperature, the leaves were further incubated in the working solution for 2 h and then decolourized twice with 2.5 g/mL chloral hydrate for 24 h. A blue colour then indicated the location of dead tissue.

### Electrolyte leakage detection

Electrolyte leakage assay was conducted as previously described (Jia *et al*., 2025). Briefly, six leaf discs 1 cm in diameter were harvested from agroinfiltrated patches of *N. benthamiana* leaves at 3 dpai. Each sample was soaked in 5 mL of sterilized distilled water for 2 h at room temperature away from light, and then replaced with fresh sterilized distilled water to remove any electrolytes that had leaked initially from the damaged cells on the edges of the leaf discs. The initial electrical conductivity was then measured using a DDS‐11A conductivity meter (INESA) and recorded as E1. The aforementioned 5 mL of sterilized water containing the leaf disc was boiled for 15 min and then cooled to room temperature. The second electrical conductivity was measured and recorded as E2. The percentage of E1 and E2 represents the degree of electrolyte leakage. Each experiment was conducted four times, and the reported values for each treatment represent the mean of these four replicates.

### Extraction of apoplast

Extraction of apoplastic fluid from treated *N. benthamiana* leaves was performed as previously described (Movahed *et al*., 2019). Briefly, whole leaves were harvested and subjected to vacuum infiltration with vesicle isolation buffer (VIB), which consisted of 20 mM MES, 2 mM CaCl_2_, and 0.1 M NaCl, adjusted to pH 6. The infiltrated leaves were gently blotted to remove any excess fluid, then placed into 30 mL syringes, and subsequently centrifuged within 50 mL conical tubes at 700g for 40 min at 4 °C to separate the apoplastic fluid.

### Protein–protein interaction analysis

For yeast two‐hybrid (Y2H) assay, various combinations of plasmids were transformed into the yeast strain NMY51 using the DUAL membrane system, as previously described (Wu *et al*., 2018). For BiFC assay, full‐length host and virus genes were fused with the N‐terminal (nYFP) or C‐terminal (cYFP) regions of YFP, respectively (Rui *et al*., 2022b). These constructs were then transferred into *A. tumefaciens* GV3101 via electroporation and infiltrated into three‐week‐old *N. benthamiana* leaves. High‐resolution imaging was performed using a Nikon A1R confocal microscope at 48 h post‐infiltration (hpi). For luciferase complementation imaging (LCI) assay, pCAMBIA1300‐cLUC or pCAMBIA1300‐nLUC vectors were fused with the protein‐coding regions of host and virus genes. After 48 h, the agrobacterium‐infiltrated leaves were collected and sprayed with 100 μM D‐Luciferin potassium salt and protected from light (Zhou *et al*., 2018). After a 10‐min incubation, the fluorescence signal on the treated leaves was captured using a low‐light cooled CDD imaging system (Amersham Imager 680, GE). For co‐immunoprecipitation (Co‐IP) assay, a nondenaturing lysis buffer was prepared by adding 0.2 g PVPP, 0.015 g DTT, and a protease inhibitor tablet to 10 mL of IP buffer, which was made by mixing 12.5 mL of 1 M Tris‐HCl, pH 7.5, 50 mL glycerol, 0.186 g EDTA, 4.383 g NaCl, and 0.1% IGEPAL® CA‐630 to make 500 mL. Approximately 0.5 g of plant samples, ground into powder in liquid nitrogen, was mixed with 1.5 mL of nondenaturing lysis buffer and agitated at 4 °C for 30 min. After centrifugation at 4 °C for 10 min at 14 000 ***g***, 100 μL of supernatant was set aside for use as the input, and the remaining supernatant was incubated with MYC‐Trap_MA beads (Chromotek, Germany) at 4 °C for 2 h. The beads were then washed five times with IP buffer, followed by immunoblot analysis.

### Peptide and chemical treatments

The mature RALF1 was synthesized by Guoping Pharmaceutical Co. Ltd. (Hefei City, China) and dissolved in sterile water to prepare a stock solution. For the preparation of the working solution, 2% dimethyl sulfoxide (DMSO) was used to dissolve Estradiol, resulting in a final concentration of 10 μM. 2% DMSO and sterilized distilled water were used to prepare working solutions of 100 mM MG132 (with DMSO serving as control) and 10 mM 3‐MA (with sterilized distilled water as control), respectively. These solutions were used for chemical treatment to inhibit the 26S proteasome and autophagy pathways (Cheng and Wang, 2017). At 2 dpai, the agro‐infiltrated regions were treated with the MG132 and 3‐MA working solutions. Leaves were incubated for an additional 8–12 h and then subjected to immunoblotting analysis.

### Statistical analysis

ImageJ software was used to process images obtained from the LUC experiment and to quantify the bands on immunoblots. For statistical analysis, GraphPad Prism 8.0 was used to determine significance using either Student's *t*‐test or one‐way ANOVA. At least three independent experiments were performed, with each experiment including at least five biological replicates or three technical replicates, unless otherwise specified.

## Author contributions

G.W. and F.Y. conceived the project; G.W., F.Y., and J.C. supervised the work; G.W. and P.R. designed the experiments; P.R. performed most of the experiments with assistance from Z.J., X.F., and T.Y.; all authors analysed the data; G.W. and P.R. wrote the manuscript.

## Conflict of interest

None declared.

## Supporting information


**Figure S1** Effect of transient expression of NbRALF1 or NbRALF23 on TuMV infection.
**Figure S2** TuMV infection up‐regulated *NbRALF1* expression.
**Figure S3** Transient silencing of NbRALF1 inhibited TuMV infection.
**Figure S4** TRV‐mediated silencing of *NbRALF1*.
**Figure S5** Systemic silencing of *NbRALF1* in *N. benthamiana* plants inhibited TuMV infection.
**Figure S6** Verification of the transgenic NbRALF1‐OE plants.
**Figure S7** Knock out of *NbRALF1* in *N. benthamiana*.
**Figure S8** NbRALF1 negatively regulates PMMoV infection.
**Figure S9** NbRALF1 negatively regulates PVX infection.
**Figure S10** NbRALF1^1–63^, NbRALF1^68–117^ and NbRALF1^C85A,C108A^ fail to inhibit TuMV infection in *N. benthamiana*.
**Figure S11** NbFER interacts with NbRALF1.
**Figure S12** NbRALF1 relies on NbFER and its phosphorylation to suppress TuMV infection.
**Figure S13** The lysine 560 residue in NbFER is crucial for its kinase activity.
**Figure S14** NbFER cooperates with NbRALF1 to inhibit TuMV replication in protoplasts.
**Figure S15** Western blot analysis of protein accumulation levels.
**Figure S16** Effect of expressing NbRALF1 or its mutants on cell death induction in *N. benthamiana*.
**Figure S17** TuMV infection did not affect the extracellular localization of NbRALF1 and its mutants.
**Figure S18** BiFC assay *in planta* to investigate the interaction domains in NbRALF1 and NbFER.
**Figure S19** TuMV‐encoded P3 and NIb proteins cannot interact with NbRALF1.
**Figure S20** TuMV VPg does not affect NbRALF1 protein accumulation.
**Figure S21** Overexpression of NbRALF1 does not affect 6K2 protein accumulation.
**Figure S22** TRV‐mediated silencing of *NbATG5, NbATG7, NbRPN10*, or *NbRPN13*.
**Figure S23** Western blot analysis of protein accumulation levels.
**Table S1** Primers used in this study.

## Data Availability

Sequence data from this article can be found in GenBank under the following accession number: TuMV (NC_002509.2). These gene sequences were derived from https://solgenomics.net and their accession numbers are as follows: *NbRALF1* (Niben101Scf06628g01013.1), *NbRALF23* (Niben101Scf00246g01001.1), and *NbFER* (Niben101Scf07619g00006.1).
