# Supplementary material for: A plant viral effector subverts FER‐RALF1 module‐mediated intracellular immunity
Source: Plant Biotechnol J. 2025 Apr 20;23(7):2734–51. doi: 10.1111/pbi.70099 (PMC12205892; doi:10.1111/pbi.70099)
Supplement: Supplementary file 1 — Figure S1 Effect of transient expression of NbRALF1 or NbRALF23 on TuMV infection. Figure S2 TuMV infection up‐regulated NbRALF1 expression. Figure S3 Transient silencing of NbRALF1 inhibited TuMV infection. Figure S4 TRV‐mediated silencing of NbRALF1. Figure S5 Systemic silencing of NbRALF1 in N. benthamiana plants inhibited TuMV infection. Figure S6 Verification of the transgenic NbRALF1‐OE plants. Figure S7 Knock out of NbRALF1 in N. benthamiana. Figure S8 NbRALF1 negatively regulates PMMoV infection. Figure S9 NbRALF1 negatively regulates PVX infection. Figure S10 NbRALF11–63, NbRALF168–117 and NbRALF1C85A,C108A fail to inhibit TuMV infection in N. benthamiana. Figure S11 NbFER interacts with NbRALF1. Figure S12 NbRALF1 relies on NbFER and its phosphorylation to suppress TuMV infection. Figure S13 The lysine 560 residue in NbFER is crucial for its kinase activity. Figure S14 NbFER cooperates with NbRALF1 to inhibit TuMV replication in protoplasts. Figure S15 Western blot analysis of protein accumulation levels. Figure S16 Effect of expressing NbRALF1 or its mutants on cell death induction in N. benthamiana. Figure S17 TuMV infection did not affect the extracellular localization of NbRALF1 and its mutants. Figure S18 BiFC assay in planta to investigate the interaction domains in NbRALF1 and NbFER. Figure S19 TuMV‐encoded P3 and NIb proteins cannot interact with NbRALF1. Figure S20 TuMV VPg does not affect NbRALF1 protein accumulation. Figure S21 Overexpression of NbRALF1 does not affect 6K2 protein accumulation. Figure S22 TRV‐mediated silencing of NbATG5, NbATG7, NbRPN10, or NbRPN13. Figure S23 Western blot analysis of protein accumulation levels. Table S1 Primers used in this study. [file PBI-23-2734-s001.docx]

**Supplementary information-Figures**

**
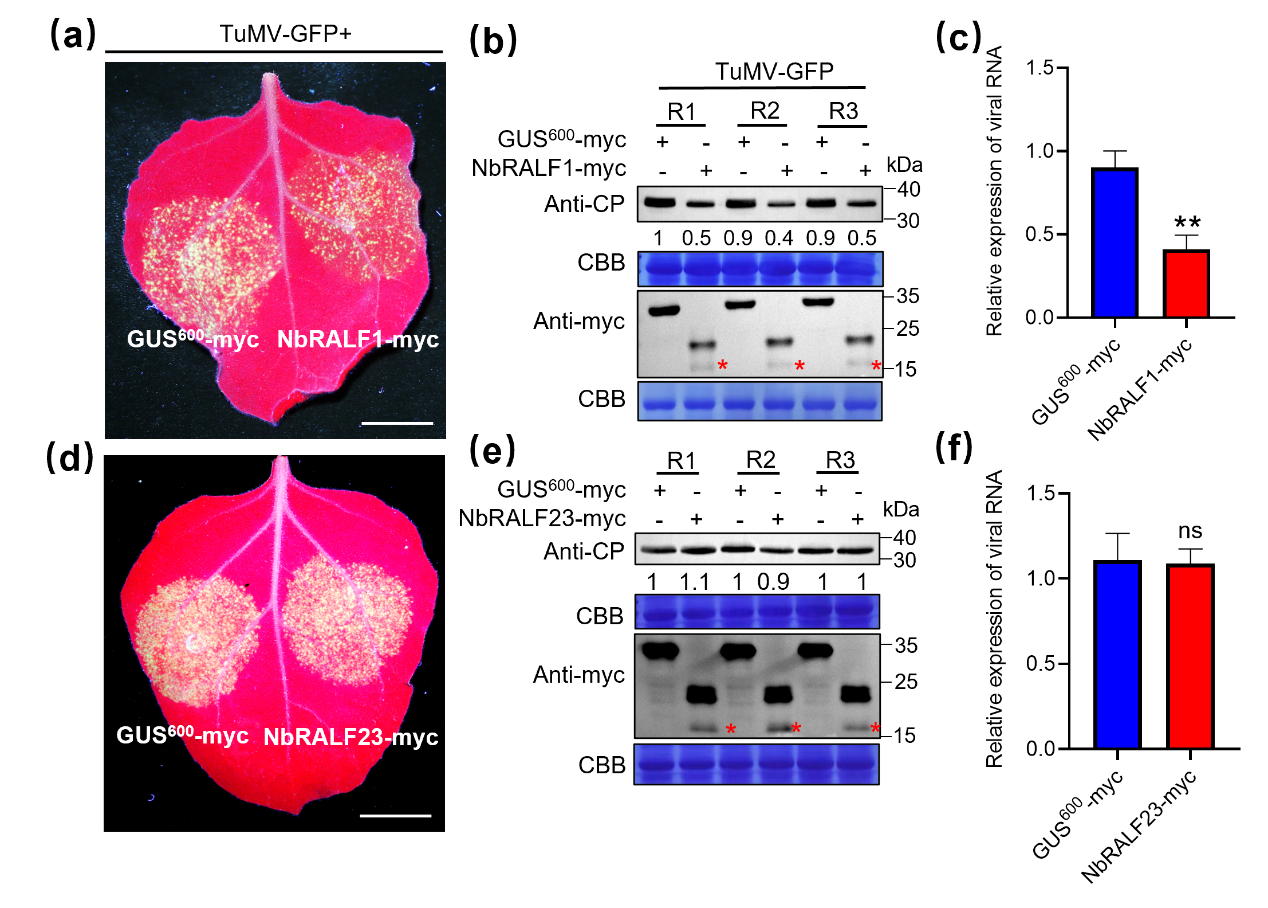
**

**Figure S1 Effect of transient overexpression of NbRALF1 or NbRALF23 on TuMV infection in *N. benthamiana***

(**a**, **d**) GFP fluorescence of TuMV-GFP infection in the inoculated leaves of *N. benthamiana* plants transiently expressing TuMV-GFP and GUS^600^-myc or NbRALF1-myc/NbRALF23-myc at 3 days post agro-infiltration (dpai) under UV light. Scale bar: 1 cm. (**b**, **e**) Western blot analysis of indicated protein accumulation levels from the samples in (a and d) at 3 dpai. The mature form of NbRALF1 and NbRALF23 with expected band size is marked with red asterisks. R1 to R3 are three biological replicates. Relative TuMV CP band intensities were quantified by ImageJ software. Coomassie Brilliant Blue (CBB) R-250-stained RuBisco large subunit served as a loading control. (**c**, **f**) RT-qPCR analysis of TuMV RNA levels from the samples in (a and d) at 3 dpai. Error bars indicate mean ± SD (n = 5 independent plants). Statistical analysis was performed using two-sided paired Student’s *t*-test (ns, non-significant; **, *P* < 0.01).


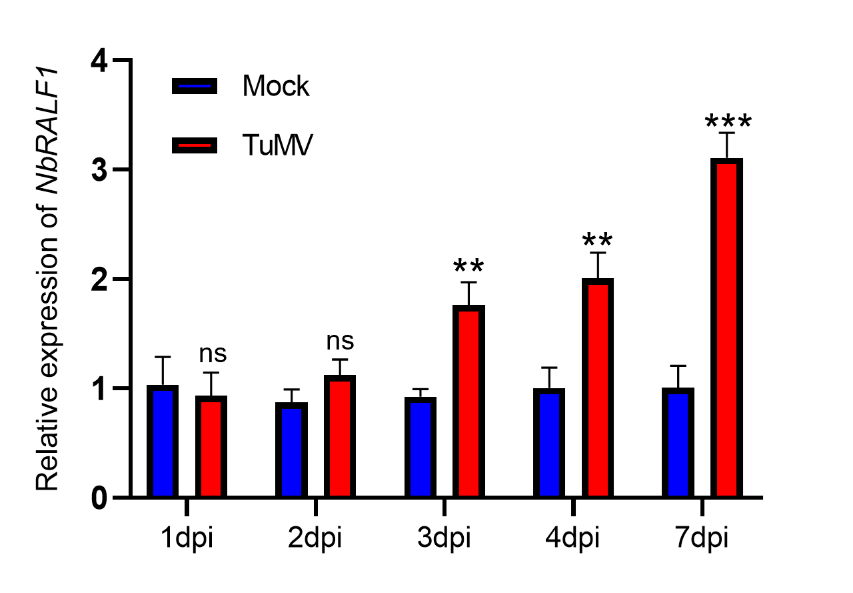


**Fig. S2 TuMV infection up-regulated *NbRALF1* expression.**

RT-qPCR analysis of *NbRALF1* RNA levels in the inoculated leaves of *N. benthamiana* leaves under TuMV-GFP infection at 1-, 2-, 3-, 4-days post-inoculation (dpi) and in the upper non-inoculated leaves of *N. benthamiana* leaves under TuMV-GFP infection at 7 dpi. Error bars indicate mean ± SD (n = 5 independent plants). Statistical analysis was performed using two-sided paired Student’s *t*-test (ns, non-significant; **, *P* < 0.01; ***, *P* < 0.001).


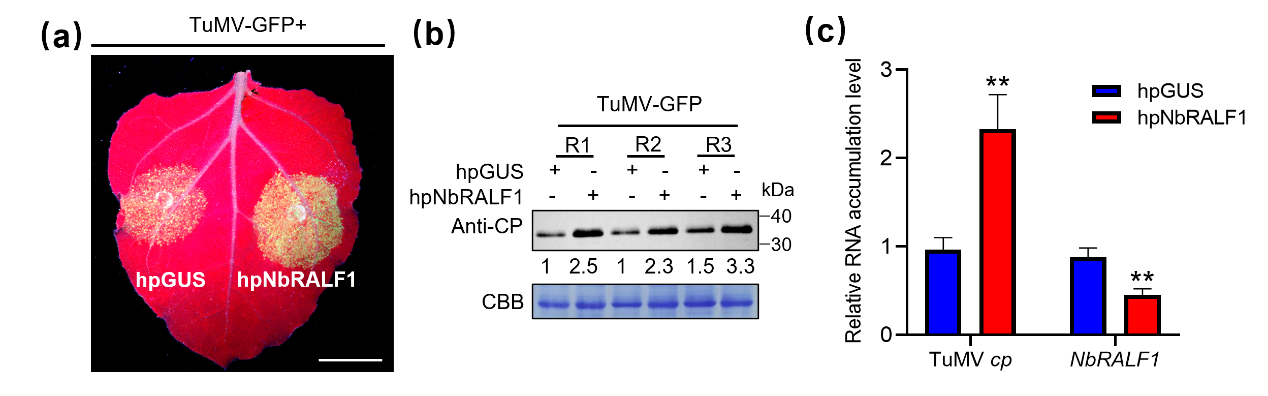


**Fig. S3 Transient silencing of *NbRALF1* inhibited TuMV infection**

(**a**) GFP fluorescence of TuMV-GFP infection in the inoculated leaves of *N. benthamiana* plants transiently expressing TuMV-GFP with either hpGUS^600^ (left) or hpNbRALF1 (right) at 3 dpai under UV light. The constructs expressing hpGUS and hpNbRALF1 were pre-agroinfiltrated one day in advance. Scale bar: 1 cm. (**b**) Western blot analysis of protein accumulation levels from the samples in (**a**) at 3 dpai. R1 to R3 are three biological replicates. Relative band intensities of TuMV CP were quantified by ImageJ software. Coomassie Brilliant Blue (CBB) R-250-stained RuBisco large subunit served as a loading control. (**c**) RT-qPCR analysis of TuMV and *NbRALF1* RNA levels from the samples in (**a**) at 3 dpai. Error bars indicate mean ± SD (n = 5 independent plants). Statistical analysis was performed using two-sided paired Student’s *t*-test (**, *P* < 0.01).

**
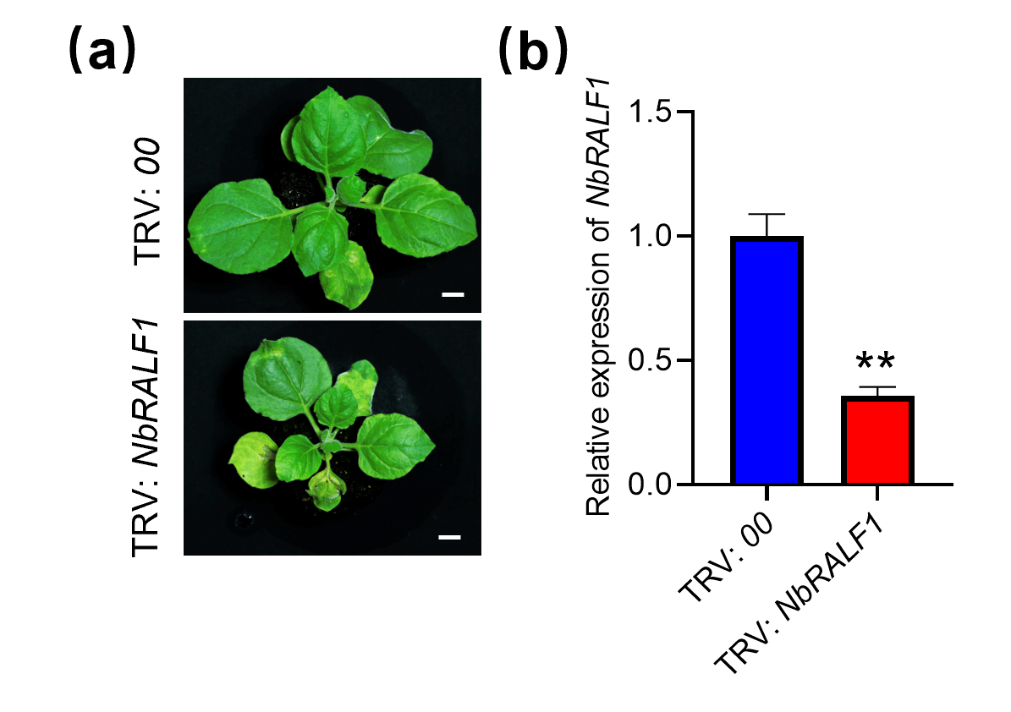
**

**Fig. S4 TRV-mediated silencing of *NbRALF1***

(**a**) Representative phenotype of *NbRALF1*-silenced *N*. *benthamiana* plants after agro-infiltration with different TRV constructs at 10 dpai. The empty vector TRV2:*00* along with TRV1 was used as negative control. Scale bar, 1 cm. (**b**) RT-qPCR analysis of *NbRALF1* RNA levels in the upper non-inoculated leaves of *N. benthamiana* plants at 10 dpai. Error bars indicate mean ± SD (n = 5 independent plants). Statistical analysis was performed using two-sided paired Student’s *t*-test (**, *P* < 0.01).


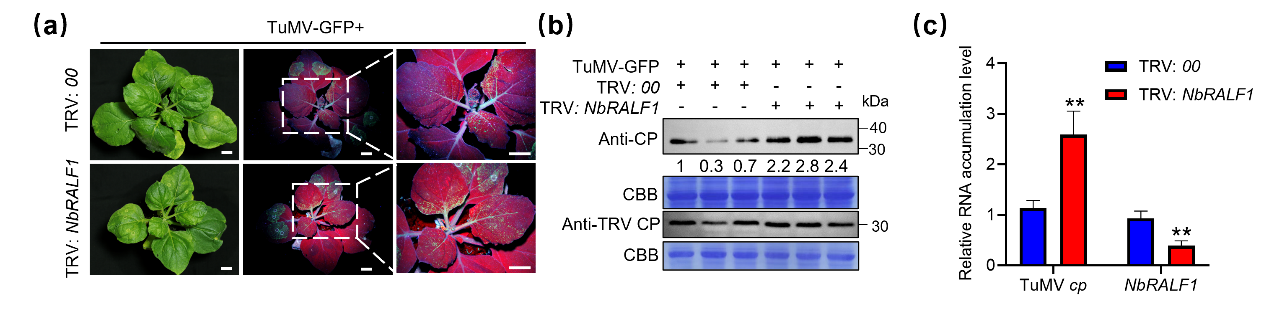


**Fig. S5 Systemic silencing of *NbRALF1* in *N. benthamiana* plants inhibited TuMV infection**

(**a**) GFP fluorescence of TuMV-GFP infection in the upper non-inoculated leaves of TRV: *00*- and TRV: *NbRALF1*- infected *N. benthamiana* plants at 6 dpai under UV light. Scale bar, 1 cm. (**b**) Western blot analysis of TuMV CP and TRV CP accumulation levels from the samples in (**a**) at 6 dpai. Relative band intensities of TuMV CP were quantified by ImageJ software. Coomassie Brilliant Blue (CBB) R-250-stained RuBisco large subunit served as a loading control. (**c**) RT-qPCR analysis of TuMV and *NbRALF1* RNA levels from the samples in (**a**) at 6 dpai. Error bars indicate mean ± SD (n = 5 independent plants). Statistical analysis was performed using two-sided paired Student’s *t*-test (**, *P* < 0.01).

**
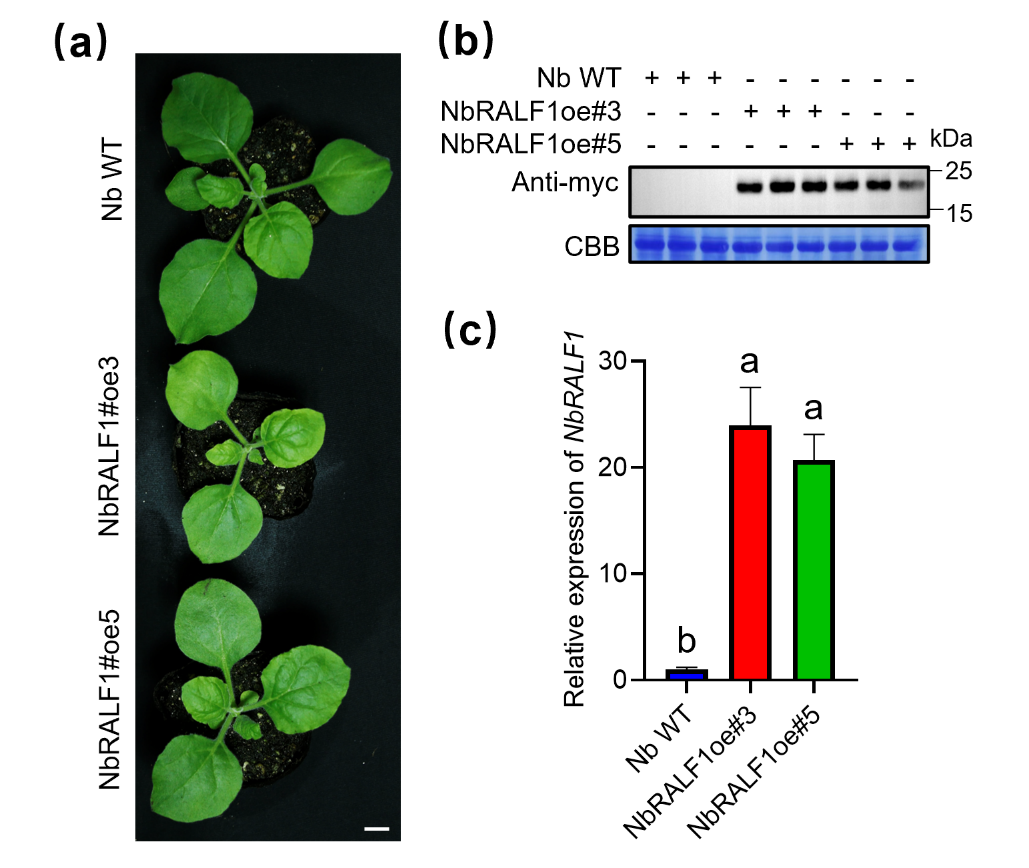
**

**Fig. S6 Verification of the transgenic NbRALF1oe plants**

(**a**) Representative phenotype of the 3-week-old NbRALF1oe *N. benthamiana* plants. Scale bar, 1 cm. (**b**) Western blot analysis of NbRALF1-myc accumulation levels from the samples in (**a**). Coomassie Brilliant Blue (CBB) R-250-stained RuBisco large subunit served as a loading control. (**c**) RT-qPCR analysis of *NbRALF1* RNA levels from the samples in (**a**). Error bars indicate mean ± SD (n = 5 independent plants). Statistical analysis was performed using One-way ANOVA with Tukey’s test; letters a–b represent statistically different groups (*P* < 0.05).

**
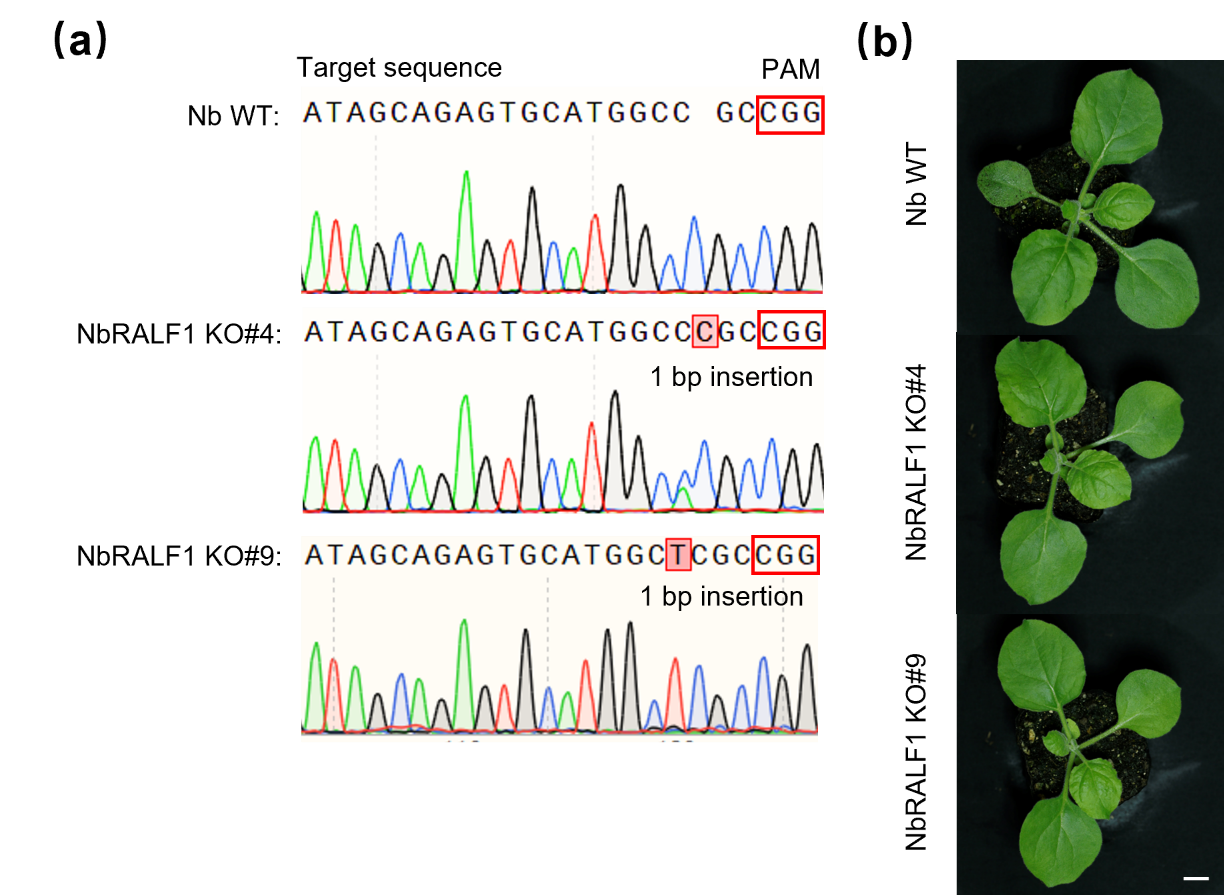
**

**Fig. S7 Knock out of *NbRALF1* in *N. benthamiana***

(**a**) DNA sequencing and alignment results showing the nucleotide insertion in the *NbRALF1* KO lines. The PAM sequence is marked with red box. (**b**) Representative phenotype of the 3-week-old *NbRALF1* KO *N. benthamiana* plants. Scale bar, 1 cm.

**
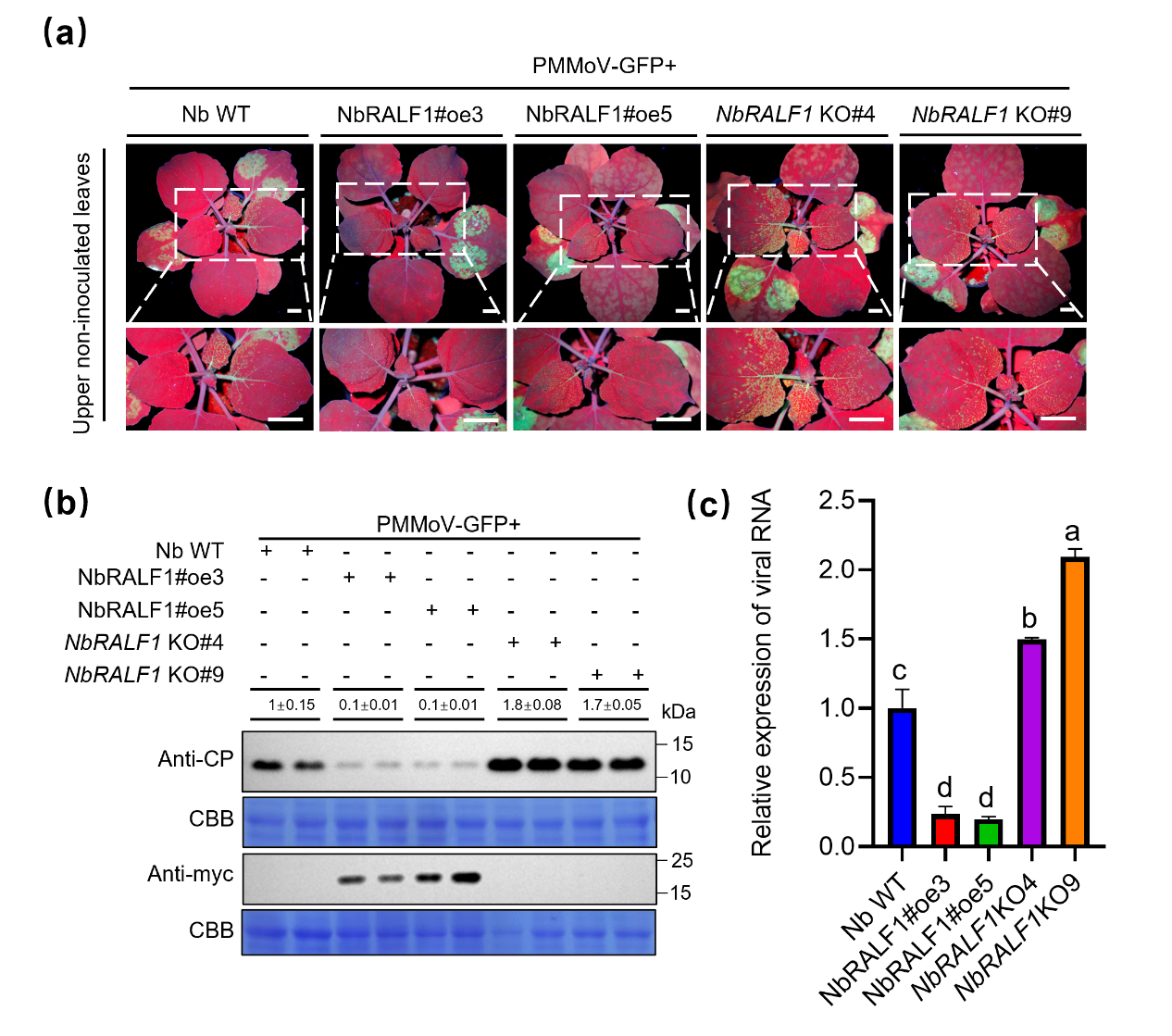
**

**Fig. S8** **NbRALF1 negatively regulates PMMoV infection**

(**a**) GFP fluorescence of PMMoV-GFP infection in the upper non-inoculated leaves of WT, RALF1oe and *RALF1*-knockout (KO) *N. benthamiana* plants at 6 dpi under UV light. Scale bar: 1 cm. (**b**) Western blot analysis of PMMoV CP accumulation levels from the samples in (**a**) at 6 dpai. Relative PMMoV CP band intensities were quantified by ImageJ software. Coomassie Brilliant Blue (CBB) R-250-stained RuBisco large subunit served as a loading control. (**c**) RT-qPCR analysis of PMMoV RNA levels from the samples in (**a**) at 6 dpai. Error bars indicate mean ± SD (n = 5 independent plants). Statistical analysis was performed using One-way ANOVA with Tukey’s test; letters a-d represent statistically different groups (*P* < 0.05).

**
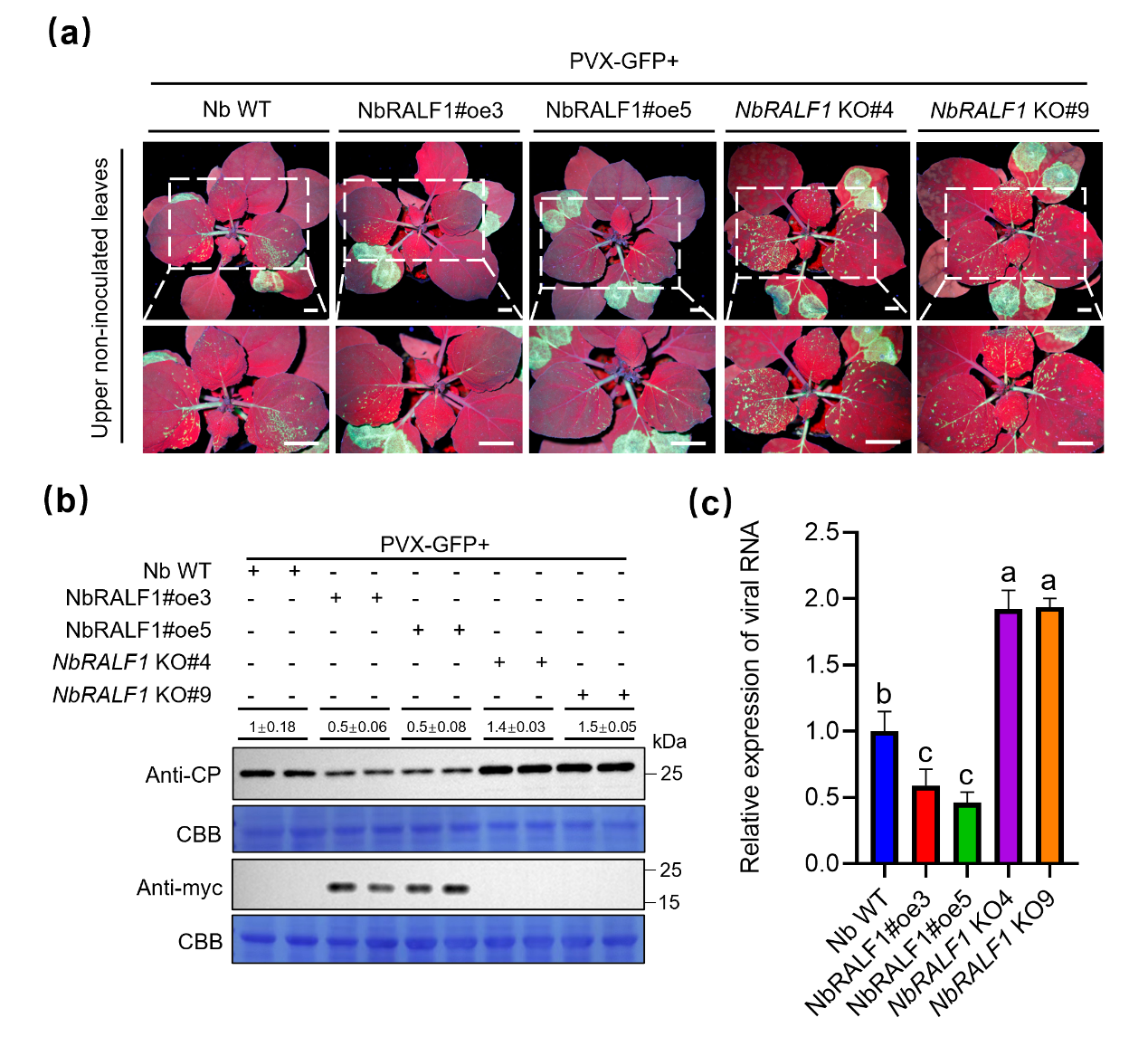
**

**Fig. S9 NbRALF1 negatively regulates PVX infection**

(**a**) GFP fluorescence of PVX-GFP infection in the upper non-inoculated leaves of WT, RALF1oe and *RALF1* KO *N. benthamiana* plants at 6 dpi under UV light. Scale bar: 1 cm. (**b**) Western blot analysis of PVX CP accumulation levels from the samples in (**a**) at 6 dpai. Relative PVX CP band intensities were quantified by ImageJ software. Coomassie Brilliant Blue (CBB) R-250-stained RuBisco large subunit served as a loading control. (**c**) RT-qPCR analysis of PVX RNA levels from the samples in (**a**) at 6 dpai. Error bars indicate mean ± SD (n = 5 independent plants). Statistical analysis was performed using One-way ANOVA with Tukey’s test; letters a-c represent statistically different groups (*P* < 0.05).

**
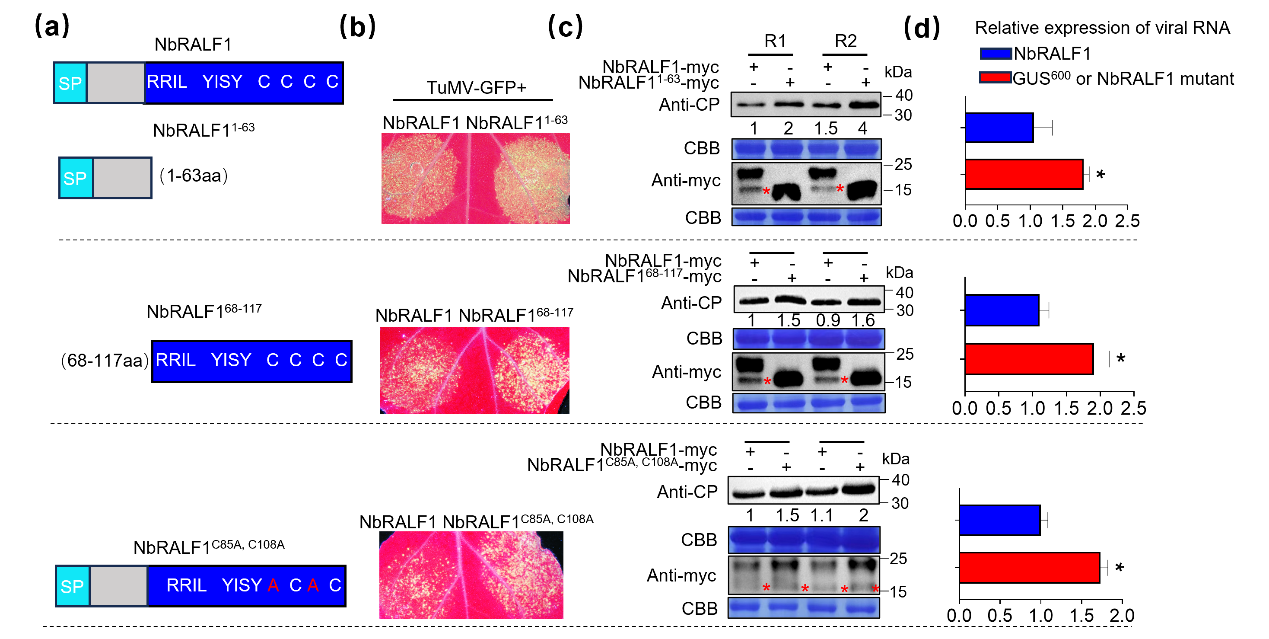
**

**Fig. S10 NbRALF1^1-63^, NbRALF1^68-117^ and NbRALF1^C85A, C108A^ fail to inhibit TuMV infection in *N. benthamiana***

(**a**) Diagram showing the construction of NbRALF1 mutants. (**b**) GFP fluorescence of TuMV-GFP infection in the inoculated leaves of *N. benthamiana* plants transiently expressing TuMV-GFP and NbRALF1 (left) or NbRALF1^1-63^/ NbRALF1^68-117^/ NbRALF1^C85A, C108A^ (right) at 3 dpai under UV light. (**c**) Western blot analysis of protein accumulation levels from the samples in (**b**) at 3 dpai. R1 and R2 are two biological replicates. The mature form of NbRALF1 with expected band size is marked with red asterisks. Relative TuMV CP band intensities were quantified by ImageJ software. Coomassie Brilliant Blue (CBB) R-250-stained RuBisco large subunit served as a loading control. (**d**) RT-qPCR analysis of TuMV RNA levels from the samples in (**b**) at 3 dpai. Error bars indicate mean ± SD (n = 5 independent plants). Statistical analysis was performed using two-sided paired Student’s *t*-test (*, *P* < 0.05).

**
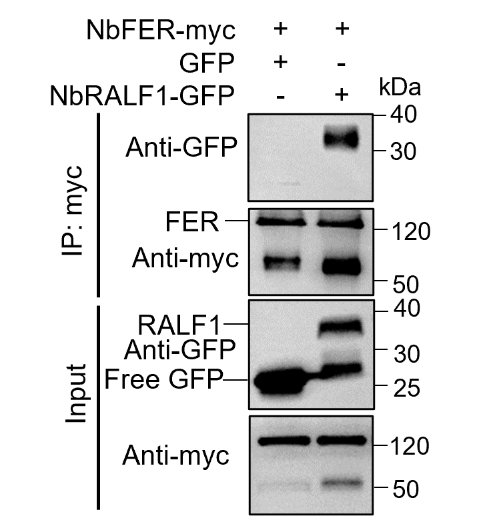
**

**Fig. S11 NbFER interacts with NbRALF1**

Results of co-IP assay showing that NbRALF1 can form complexes with NbFER in *N*. *benthamiana* cells. Different cell lysates were co-immunoprecipitated with anti-myc beads, separated by SDS-PAGE and immunoblotted with anti-myc antibody, or anti-GFP antibody.

**
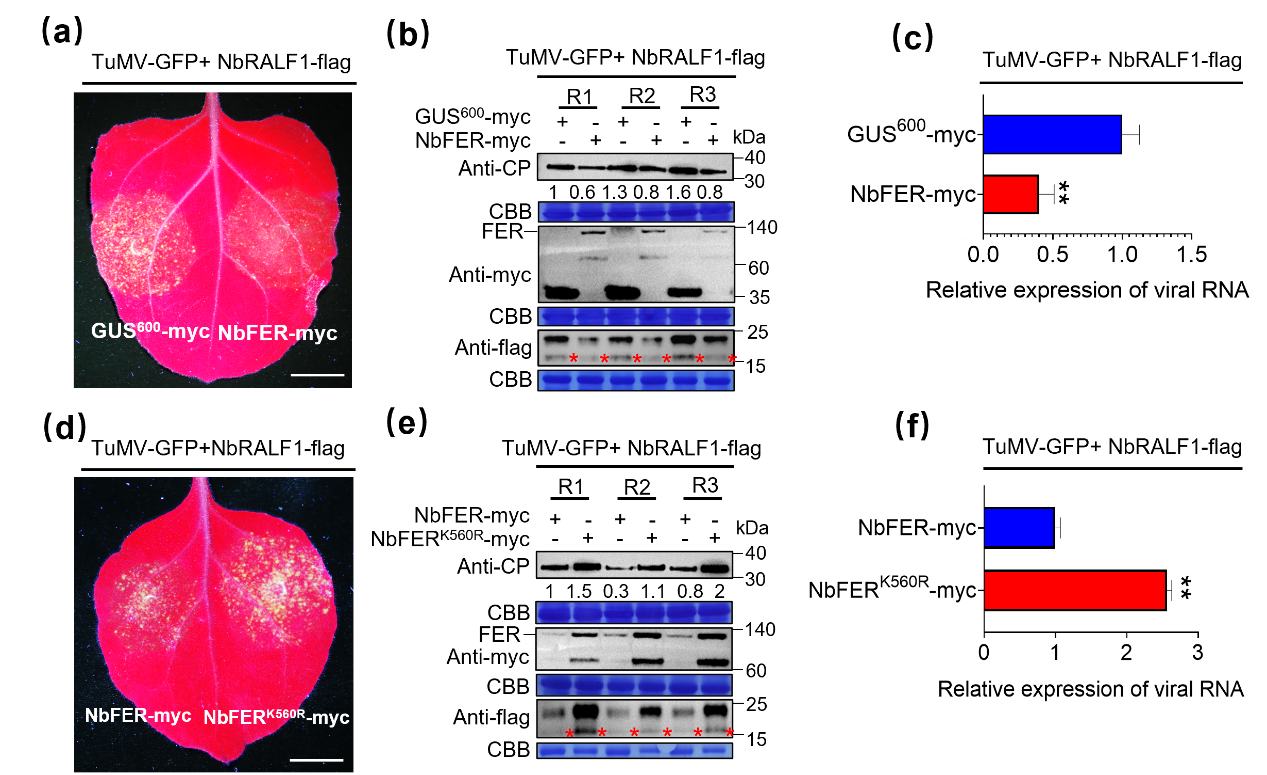
**

**Fig. S12 NbRALF1 relies on NbFER and its phosphorylation to suppress TuMV infection.**

(**a**) GFP fluorescence of TuMV-GFP infection in the inoculated leaves of *N. benthamiana* plants transiently expressing TuMV-GFP, NbRALF1-flag, together with GUS^600^-myc (left) or NbFER-myc (right) at 3 dpai under UV light. (**b**) Western blot analysis of protein accumulation levels from the samples in (**a**) at 3 dpai. (**c**) RT-qPCR analysis of TuMV RNA levels from the samples in (**a**) at 3 dpai. (**d**) GFP fluorescence of TuMV-GFP infection in the inoculated leaves of *N. benthamiana* plants transiently expressing TuMV-GFP, NbRALF1-flag, along with NbFER-myc (left) or NbFER^K560R^-myc (right) at 3 dpai under UV light. NbFER^K560R^ is a kinase-inactive mutant. (**e**) Western blot analysis of protein accumulation levels from the samples in (**d**) at 3 dpai. (**f**) RT-qPCR analysis of TuMV RNA levels from the samples in (**d**) at 3 dpai. For data in panels (**b**) and (**e**), the mature form of NbRALF1 with expected band size is marked with red asterisks. R1 to R3 are three biological replicates. Relative TuMV CP band intensities were quantified by ImageJ software. Coomassie Brilliant Blue (CBB) R-250-stained RuBisco large subunit served as a loading control. For data in panels (**c**) and (**f**), error bars indicate mean ± SD (n = 5 independent plants). Statistical analysis was performed using two-sided paired Student’s *t*-test (**, *P* < 0.01).

**
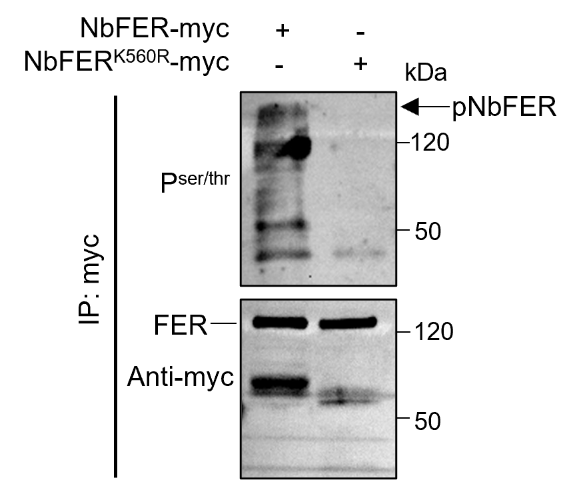
**

**Fig. S13 The lysine 560 residue in NbFER is crucial for its kinase activity**

Western blot analysis of phosphorylated NbFER (pNbFER) in the leaves of *N. benthamiana* plants. Different cell lysates were immunoprecipitated with anti-myc beads, separated by SDS-PAGE and immunoblotted with anti-myc antibody, or anti-Phosphoserine/threonine antibody.


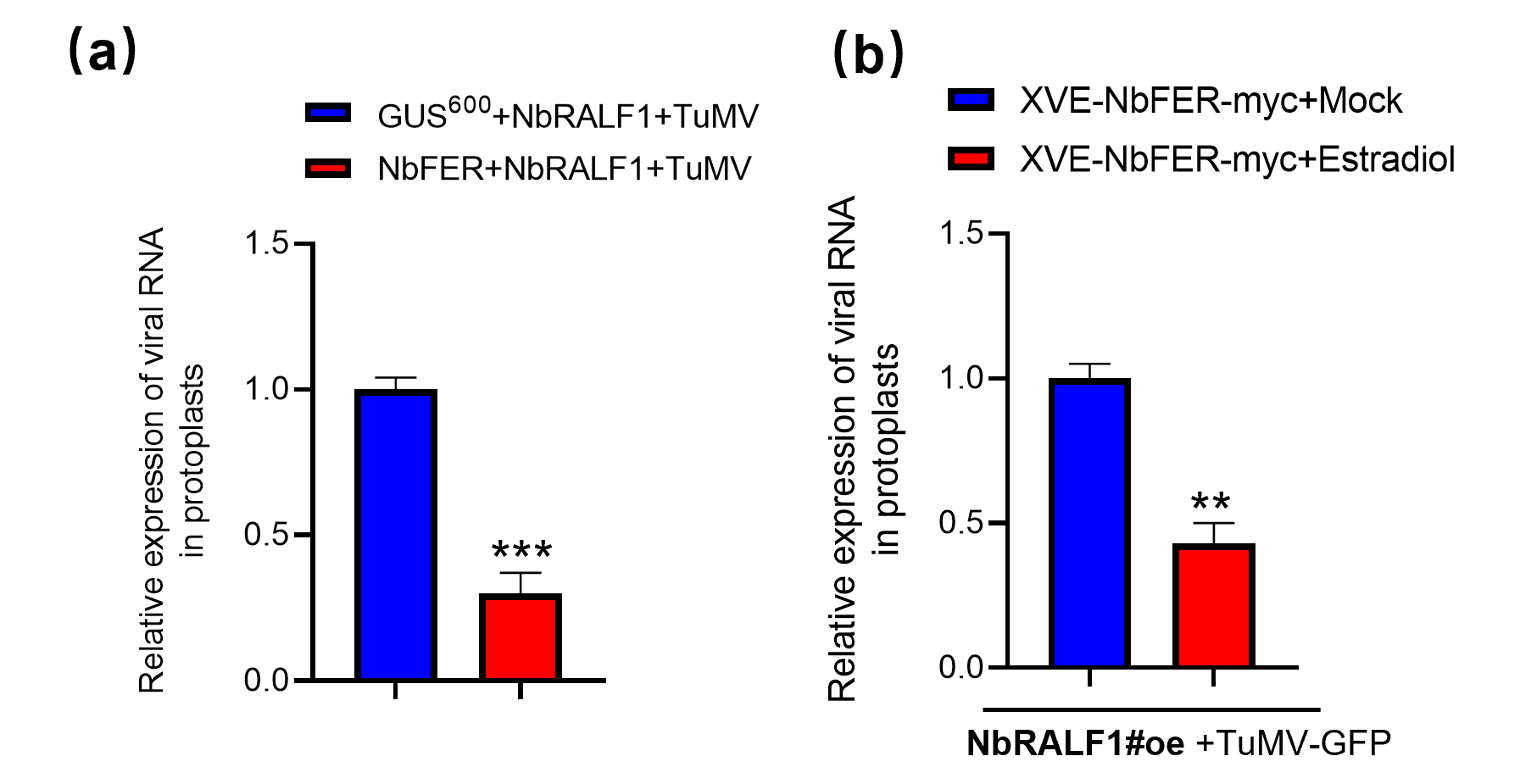


**Fig. S14 NbFER cooperates with NbRALF1 to inhibit TuMV replication in protoplasts.**

(**a**) RT-qPCR analysis of TuMV RNA levels in wild-type *N. benthamiana* protoplasts co-transfected with TuMV-GFP, NbRALF1-myc, and either NbFER-myc or GUS^600^-myc plasmids, performed 16 hours post-transfection. (**b**) RT-qPCR analysis of TuMV RNA levels in NbRALF1oe *N. benthamiana* protoplasts co-transfected with TuMV-GFP and XVE-NbFER-myc plasmids, conducted 16 hours post-transfection. Inducible expression of NbFER was achieved by adding 10 μM estradiol, with 2% DMSO serving as the control. Error bars indicate mean ± SD (n = 3 independent protoplasts). Statistical analysis was performed using two-sided paired Student’s *t*-test (**, *P* < 0.01; ***, *P* < 0.001).

**
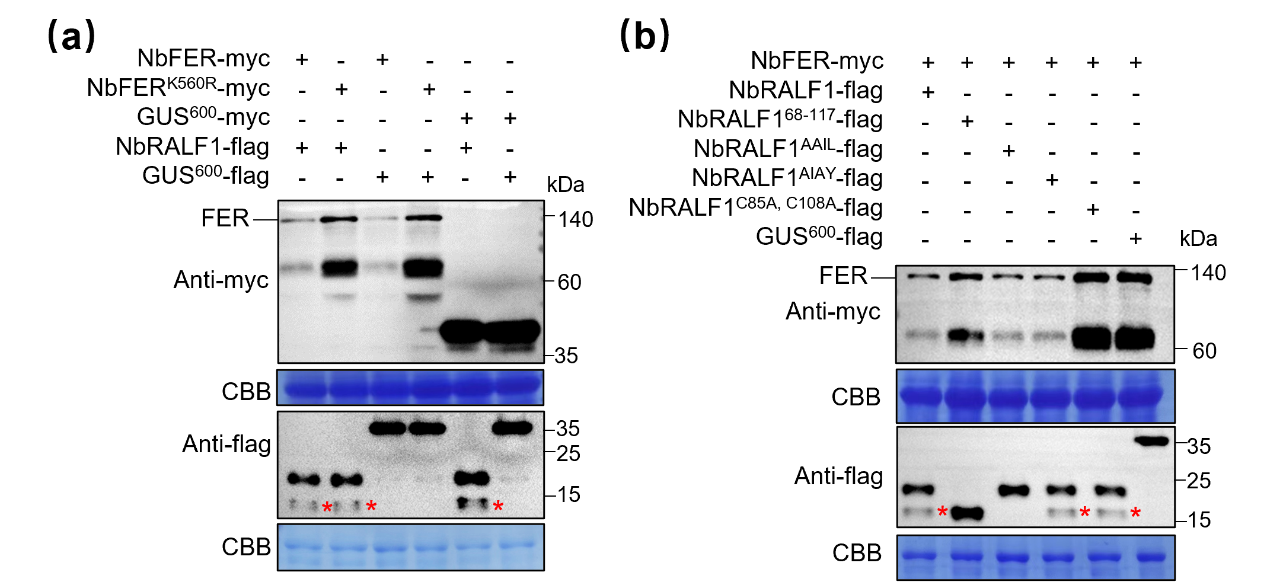
**

**Fig. S15 Western blot analysis of protein accumulation levels**

The samples from Fig. 4j (**a**) and Fig. 4k (**b**) were collected at 3 dpai for Western blotting analysis. The mature form of NbRALF1 with expected band size is marked with red asterisks. Coomassie Brilliant Blue (CBB) R-250-stained RuBisco large subunit served as a loading control.


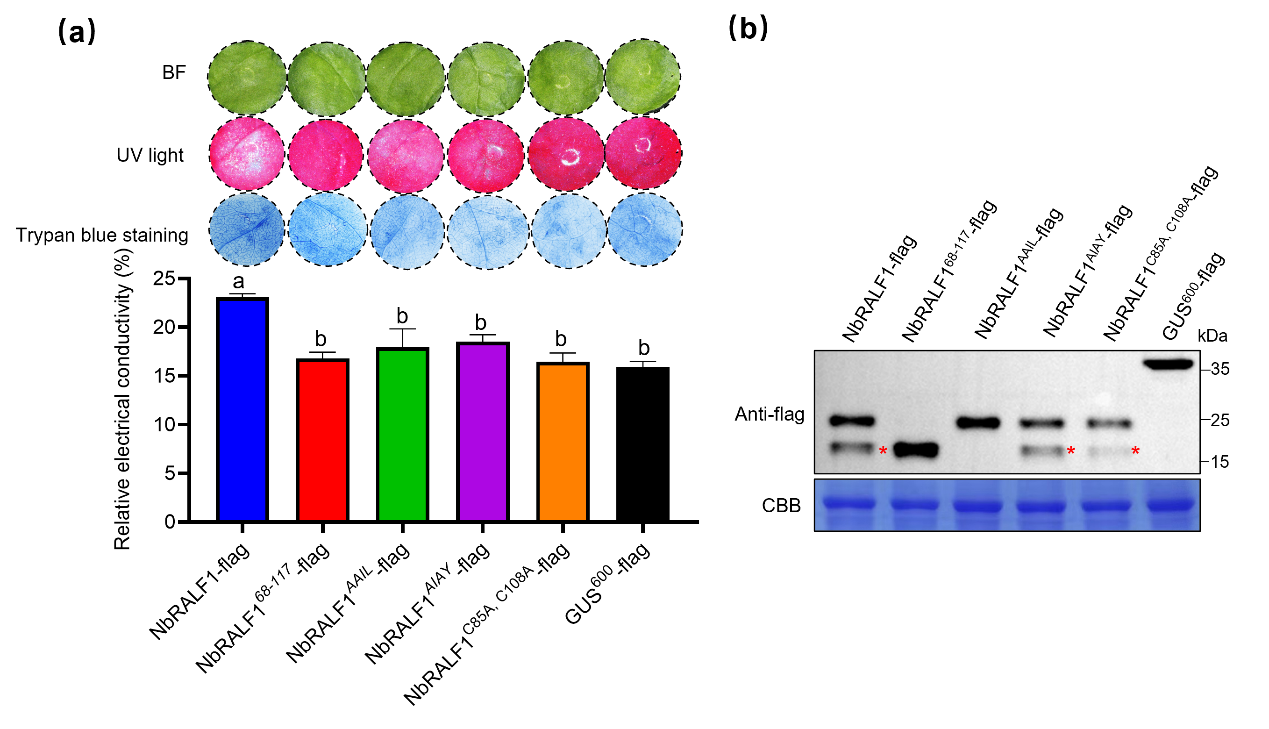


**Fig. S16 Effect of expressing NbRALF1 or its mutants on cell death induction in *N. benthamiana***

(**a**) Induction of cell death in *N. benthamiana* leaf tissues expressing different constructs as indicated. Photographs were captured under bright light and UV light at 3 dpai. Cell death was observed by staining with trypan blue and quantified by measuring electrolyte leakage. Error bars indicate mean ± SD (n = 5 independent plants). Statistical analysis was performed using One-way ANOVA with Tukey’s test; letters a-b represent statistically different groups (*P* < 0.05). (**b**) Western blot analysis of the protein accumulation from the samples in (a). The mature form of NbRALF1 with expected band size is marked with red asterisks. Coomassie Brilliant Blue (CBB) R-250-stained RuBisco large subunit served as a loading control.

**
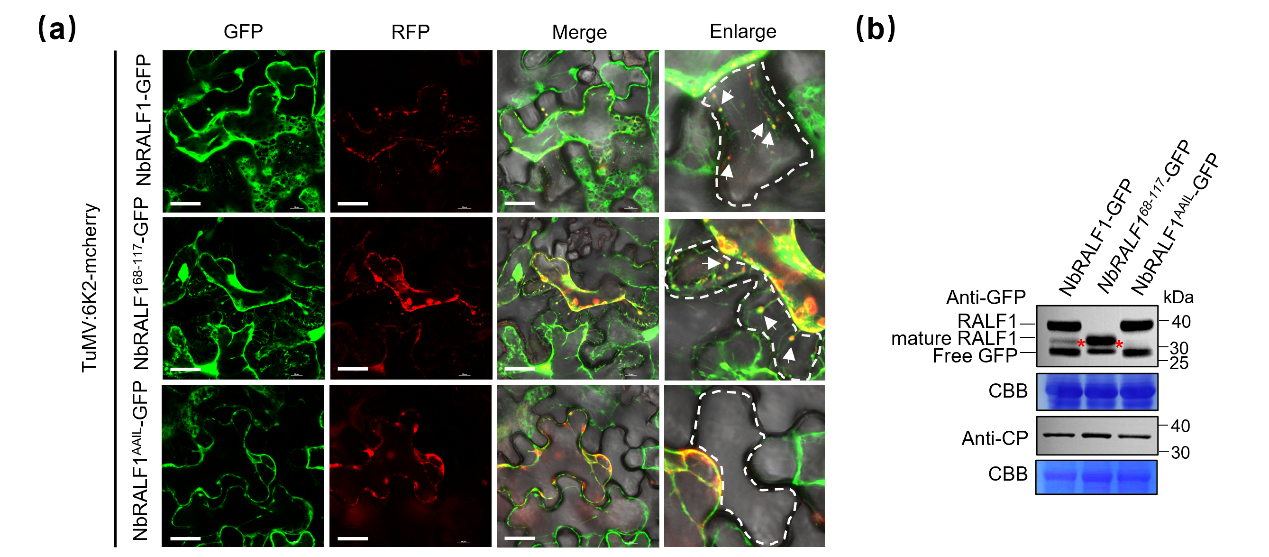
**

**Fig. S17 TuMV infection did not affect the extracellular localization of NbRALF1 and its mutants.**

(**a**) Plasmolysis assay for apoplast localization of NbRALF1 and its mutants NbRALF1^68-117^ and NbRALF1^AAIL^ under TuMV infection. NbRALF1-GFP, NbRALF1^68-117^-GFP or NbRALF1^AAIL^-GFP were transiently co-expressed with a recombinant TuMV: 6K2-mcherry infectious clone in *N. benthamiana* leaves, and then infiltrated with 10% NaCl at 2 dpai. Plasmolyzed cells in the leaf were examined using confocal microscopy. Representative apoplast localization signals and regions are indicated with white arrows and dashed lines, respectively. Scale bar represents 20 μm. (**b**) Western-blot analysis with anti-TuMV CP and anti-GFP antibodies of total protein extractions from (**a**). The mature form of NbRALF1 with expected band size is marked with red asterisks. Coomassie Brilliant Blue (CBB) R-250-stained RuBisco large subunit served as a loading control.

**
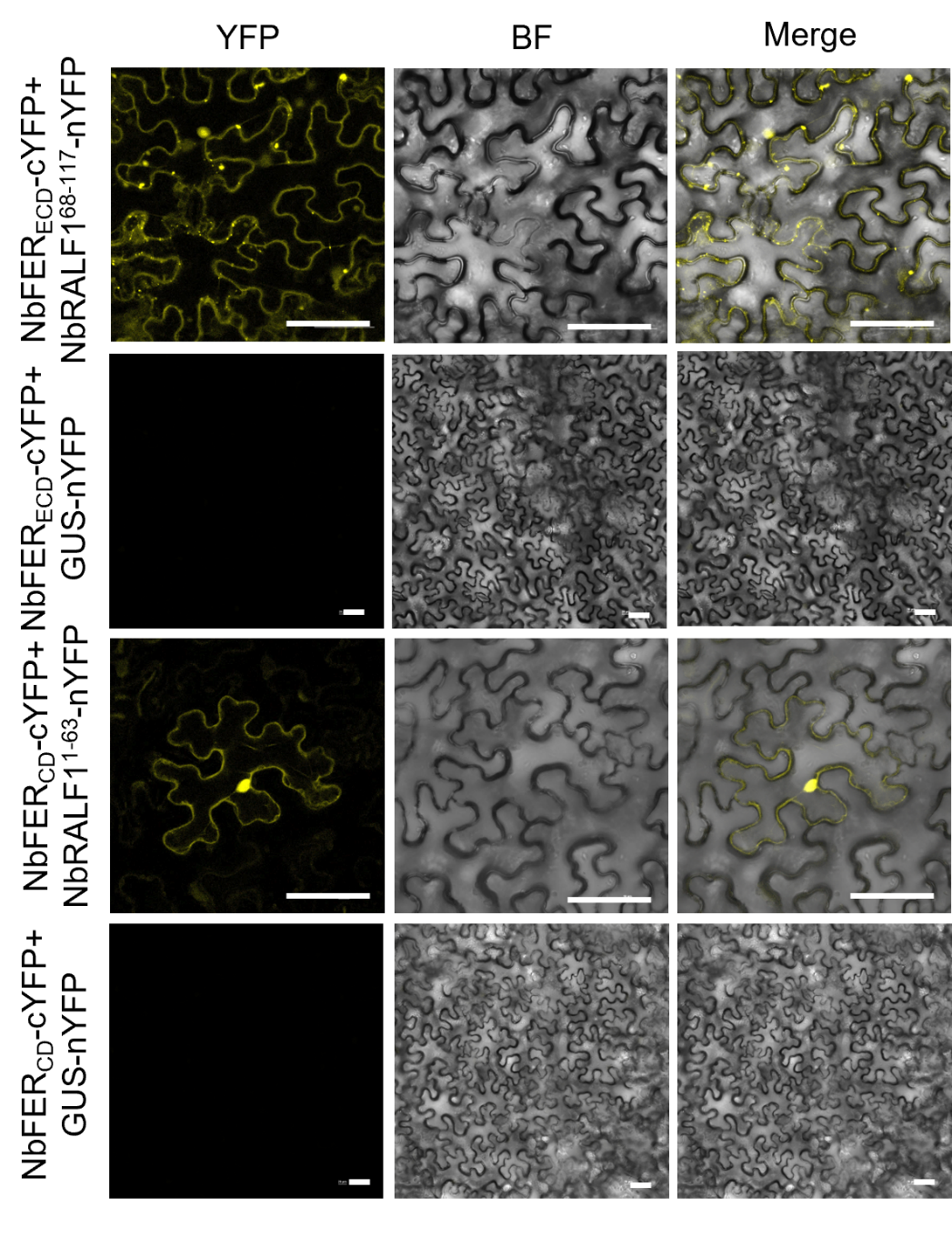
**

**Fig. S18 BiFC assay *in planta* to investigate the interaction domains in NbRALF1 and NbFER.**

Images from a BiFC assay *in planta* to confirm the interactions detected by co-IP. The interactions between NbRALF1^68-117^ and NbFER_ECD_, NbRALF1^1-63^ and NbFER_CD_ were confirmed in *N*. *benthamiana* cells. Pictures were taken at 2 dpai. The YFP field and overlay of YFP with bright field are shown. BF, bright field, Scale bar = 50 μm.

**
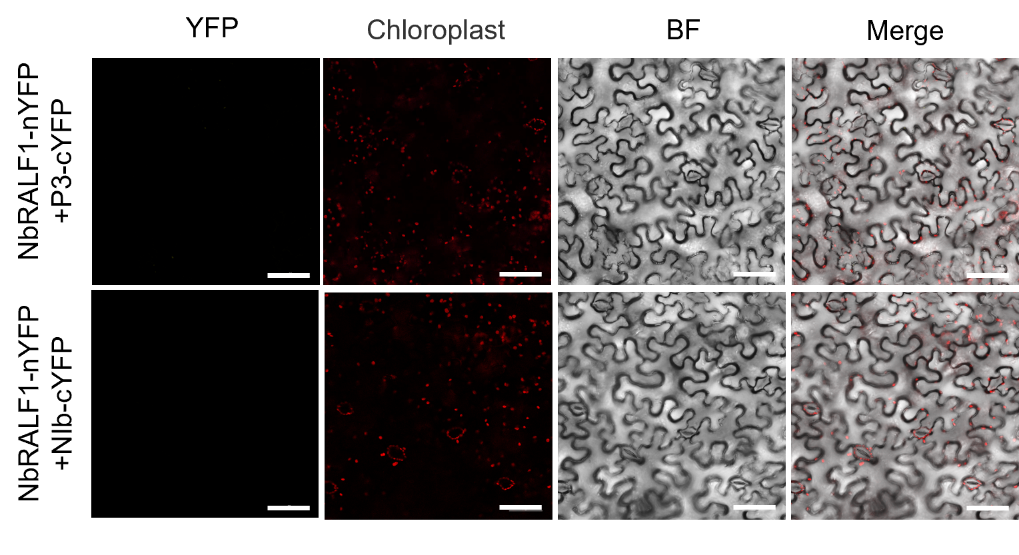
**

**Fig. S19 TuMV-encoded P3 and NIb show no interaction with NbRALF1**

Images from a BiFC assay *in planta* to investigate the interactions between NbRALF1 and TuMV P3 or NIb. Pictures were taken at 2 dpai. The YFP field and overlay of YFP with bright field are shown. BF, bright field; Chl, autofluorescent chloroplasts. Scale bar = 50 μm.


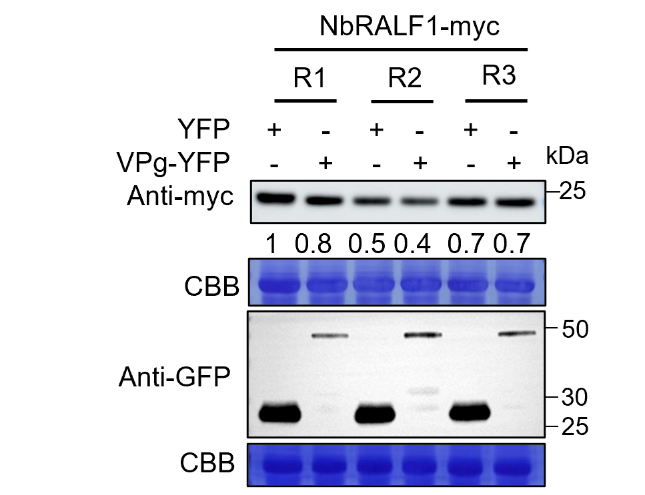


**Fig. S20 TuMV VPg does not affect NbRALF1 protein accumulation**

Western blot analysis of NbRALF1-myc, YFP, and VPg-YFP accumulation levels in the inoculated leaves of *N. benthamiana* plants transiently co-expressing NbRALF1 with YFP or VPg-YFP at 2 dpai. R1 to R3 are three biological replicates. Relative band intensities of NbRALF1-myc were quantified by ImageJ software. Coomassie Brilliant Blue (CBB) R-250-stained RuBisco large subunit served as a loading control.

**
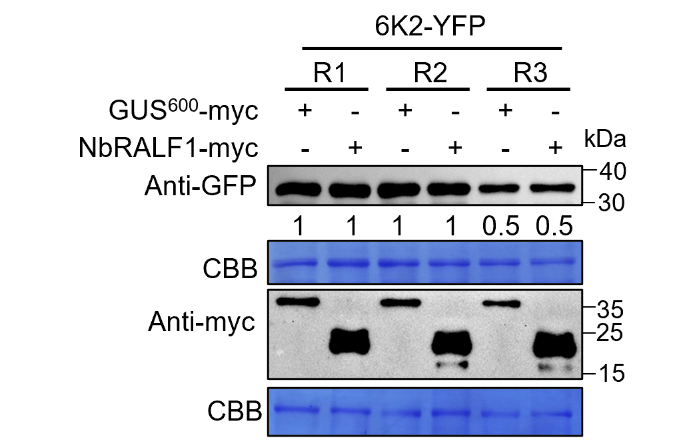
**

**Fig. S21 Overexpression of NbRALF1 does not affect 6K2 protein accumulation.**

(**a**) The effect of NbRALF1-myc overexpression on 6K2-YFP protein accumulation. GUS^600^-myc was used as a control. 6K2-YFP was co-expressed with either NbRALF1-myc or GUS^600^-myc in two patches of the same wild-type *N. benthamiana* leaf. Samples were harvested at 2 dpai for Western blotting assay with anti-c-myc and anti-GFP monoclonal antibodies. R1 to R3 are three biological replicates. The relative 6K2-YFP band intensities were quantified by ImageJ software. Coomassie Brilliant Blue (CBB) R-250-stained RuBisco large subunit served as a loading control.

**
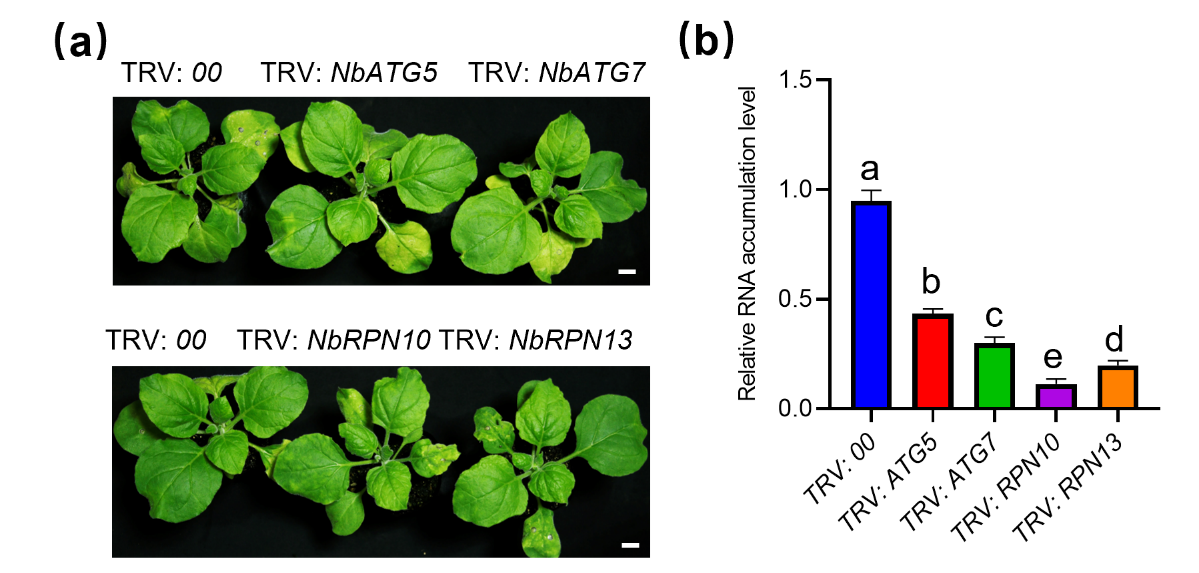
**

**Fig. S22 TRV-mediated silencing of *NbATG5*, *NbATG7*, *NbRPN10*, or *NbRPN13***

(**a**) Representative images of *NbATG5*, *NbATG7*, *NbRPN10*, and *NbRPN13*-silenced *N. benthamiana* plants after agro-infiltration with different TRV constructs at 10 dpai. Scale bar, 1 cm. (**b**) RT-qPCR analysis of *NbATG5*, *NbATG7*, *NbRPN10*, and *NbRPN13* RNA levels from the samples in (a). Error bars indicate mean ± SD (n = 5 independent plants). Statistical analysis was performed using One-way ANOVA with Tukey’s test; letters a-e represent statistically different groups (*P* < 0.05).

**
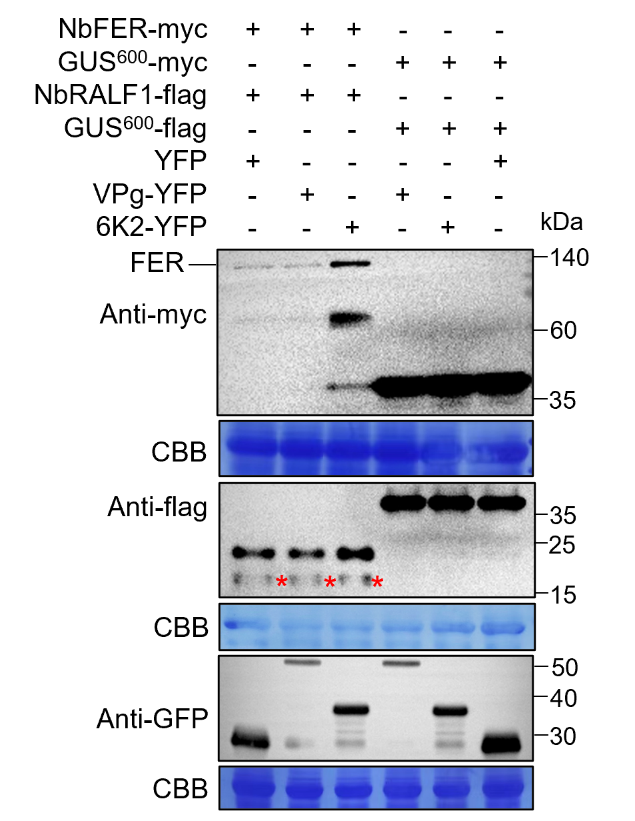
**

**Fig. S23 Western blot analysis of protein accumulation levels**

Samples from Fig. 7g were collected for Western blotting analysis at 3 dpai. The mature form of NbRALF1 with expected band size was marked with red asterisks. Coomassie Brilliant Blue (CBB) R-250-stained RuBisco large subunit served as a loading control.

**Table S1 Primers used in this study**

| **Primer names** | **Sequences (5’ to 3’)** | **Notes** |
| --- | --- | --- |
| lic-NbRALF1-F | CgACgACAAgACCgTCAccATGGCGAAGTCGTTTAGCTC | For Ligation independent cloning. |
| lic-NbRALF1-R | gAggAgAagAgCCgTcgACTCCGGCAACGAGTAATAG |  |
| lic-NbRALF23-F | CgACgACAAgACCgTCAccATGGCGATGGTGAAAGTGAAC |  |
| lic-NbRALF23-R | gAggAgAagAgCCgTcgACGGCGGCAGCGCGTGAT |  |
| Lic-NbFER-F | CgACgACAAgACCgTCAccATGACAGAAGGCAGTAAATTC |  |
| Lic-NbFER-R | gAggAgAagAgCCgTcgGCGTCCTTTTGGATTCATGAT |  |
| LIC-AtPEN1-F | CgACgACAAgACCgTCAccATGAACGATTTGTTTTCCAGCTC |  |
| LIC-AtPEN1-R | gAggAgAagAgCCgTcgACGCAATAGACGCCTTGC |  |
| LIC-NbRALF1^1-64^-F | CgACgACAAgACCgTCAccATGGCGAAGTCGTTTAGCTC | For overlapping PCR to generate NbRALF1 mutants |
| LIC-NbRALF1^1-64^-R | gAggAgAagAgCCgTcgGTTGCTCTCTGAATCCATTTCG |  |
| LIC-NbRALF1^68-117^-F | CgACgACAAgACCgTCAccATGGCAACTGATGATTACATAAGC |  |
| LIC-NbRALF1^AAIL^-F | AGCAACGCAGCCATTTTAGCAACTGATGATTACATAAGC |  |
| LIC-NbRALF1^AAIL^-R | TAAAATGGCTGCGTTGCTCTCTGAATCCATTTCGAATTCAC |  |
| LIC-NbRALF1^AIAY^-F | GATGATGCCATAGCCTATGGTGCTCTGCAGAGAAAC |  |
| LIC-NbRALF1^AIAY^-R | ATAGGCTATGGCATCATCAGTTGCTAAAATGCGTCGG |  |
| LIC-NbRALF1^C85A,C108A^-R | gAggAgAagAgCCgTcgACTCCGGCAACGAGTAATAGCACTGgcGCCACGGGTATAAGGATTAGCTTCAGCACCTGTTTGGCAGTTATAATAGGAAGCACCCCTTCTAGAAGCGGGTACAGTGTTTCTCTG |  |
| NbFER-K560R-F | AGTTGCCATCAGACGTGGAA | For generation of NbFFER non-phosphorylated mutant |
| NbFER-K560R-R | TTCCACGTCTGATGGCAACT |  |
| LIC-NbFER (MLD)-F | CgACgACAAgACCgTCAccATGCTGTTGAATTGTGGAGGTCC | For generation of NbFER truncated mutants |
| LIC-NbFER (MLD)-R | gAggAgAagAgCCgTcgGGGATGGAGGGCAAGCCA |  |
| LIC-NbFER (Kinase)-F | CgACgACAAgACCgTCAccATGATTCTTATCGGTTTGGTTGTA |  |
| LIC-NbFER (Kinase)-R | gAggAgAagAgCCgTcgGCGTCCTTTTGGATTCATGA |  |
| NbRALF1-crispr-F | TCGTTTAGCTCCACTTTC | For PCR genotyping |
| NbRALF1-crispr-R | ATGACCACGATCACTTTA |  |
| qNbRALF1-F | GGATTCAGAGAGCAACCGAC | For amplifying  NbRALF1 (Niben101Scf06628g01013.1 ). |
| qNbRALF1-R | ACTCCGGCAACGAGTAATAG |  |
| qNbFER-F | ATGACAGAAGGCAGTAAATTCTG | For amplifying NbFER (Niben101Scf07619g00006.1 ). |
| qNbFER-R | GACTTGCTACCCTTCAAATATGT |  |
| qTuMV CP-F | TGGCTGATTACGAACTGACG | For detecting the TuMV isolate (NC_002509.2). |
| qTuMV CP-R | CTGCCTAAATGTGGGTTTGG |  |
| qNbActin-F | GGGATGTGAAGGAGAAGTTGGC | For detecting *NbACT* (AY179605). |
| qNbActin-R | ATCAGCAATGCCCGGGAACA |  |
| LIC-NbATG5-F | cgacgacaagaccgtTGGGAAGTAAAGGGGCAG | Primers previously described by Ji *et al*., 2021. |
| LIC-NbATG5-R | ggaggagaagagccgtcgACGTTCAGGTTCTGCACA |  |
| LIC-NbATG7-F | cgacgacaagaccgtTGGCGGATAGTGGAAGAGGA |  |
| LIC-NbATG7-R | ggaggagaagagccgtcgCAACGTGTTTGTATTGAGAA |  |
| LIC-NbRPN10-F | cgacgacaagaccgtcaccTTTCAATGGAGGAGGAAA |  |
| LIC-NbRPN10-R | gaggagaagagccgtcgCTTCTTCTCTTCTTTCTGTTCC |  |
| LIC-NbRPN13-F | cgacgacaagaccgtcaccGAGTTCCCTGGTGAAGA |  |
| LIC-NbRPN13-R | gaggagaagagccgtcgTGGGATCAATTCCAAACTGA |  |
| qNbATG5-F | AAGCTCATACGCATTCAGGG |  |
| qNbATG5-R | GCTTCGGACCTTTGCTACCT |  |
| qNbATG7-F | CCAGCAGTGGAAGCAGAAGGTCTT |  |
| qNbATG7-R | CCAGCAGTGGAAGCAGAAGGTCTT |  |
| qNbRPN10-F | CCAGCAGTGGAAGCAGAAGGTCTT |  |
| qNbRPN10-R | CCAGCAGTGGAAGCAGAAGGTCTT |  |
| qNbRPN13-F | CCAGCAGTGGAAGCAGAAGGTCTT |  |
| qNbRPN13-R | CCAGCAGTGGAAGCAGAAGGTCTT |  |
| qNbMEKK4-F | GTGATTGGGCTAGTCTTATGTGC | Primers designed from the *N. benthamiana* reference genome Nb HZ version 1, available at <https://lifenglab.hzau.edu.cn/Nicomics> |
| qNbMEKK4-R | GTGGTAGTGGCTGGTCTATTC |  |
| qNbMEKK5-F | ACACGGACCTAAATACAGGCC |  |
| qNbMEKK5-R | CCGTCCACCTTCTAGAAGGATC |  |
| qNbMKS1-F | CCATAGAGAACACAAGGACACC |  |
| qNbMKS1-R | GGTGGTATAGGTGGAAGTGAAGAC |  |
| qNbJAR1-F | AGCTGATCCGGGACACTATGTC |  |
| qNbJAR1-R | GGCTAACTGCACCTCCTAATCC |  |
| qNbTGA-F | GTTCGCAACCTCACCCAATCC |  |
| qNbTGA-R | TCTTGGCGAAGATGATCTGCC |  |
| qNbMAPK3-F | TACGCTTGTTAACCGAGCTTCTC |  |
| qNbMAPK3-R | GCAAGGTACGGATGATCTAATGC |  |
| qNbMAPK6-F | GATGTATGGTCAGTGGGTTGC |  |
| qNbMAPK6-R | CGACGGTAAAGAGGAAGTTGTC |  |
| qNbHIN-F | CGGTCCACCCGAAGTCAA |  |
| qNbHIN-R | ACGCCGTCGATGAAGTAGTCA |  |
| qNbNPR1-F | TGAGATTCTGGAGCAAGCA |  |
| qNbNPR1-R | GTTGTCCTCTGTGCGTTGA |  |
| qNbPR1-F | ACAAGACTATTTGGATGCCC |  |
| qNbPR1-R | TCTCAACAGCCTTAGCAGC |  |
